# An open-source bio-logger for studying cetacean behavior and communication

**DOI:** 10.1371/journal.pone.0337093

**Published:** 2025-12-11

**Authors:** Daniel M. Vogt, Joseph DelPreto, Michael Salino-Hugg, Matthew R. Cummings, Michael A. Bell, Aidan Kenny, Peter Malkin, Alyssa M. Hernandez, Andrew L. Wright, Molly A. Duncan, Matthew R. Davidsen, Kaveet Grewal, Saksham Ahuja, Kelly Ostrom, Matthew Garcia, Phillip Shahviri, Chanuth Weeraratna, Hannah Zink, Stefano Pagani, Germain Meyer, Roee Diamant, Daniela Rus, David F. Gruber, Shane Gero, Robert J. Wood

**Affiliations:** 1 Harvard John A. Paulson School of Engineering and Applied Sciences, Harvard University, Cambridge, Massachusetts, United States of America; 2 Project CETI, New York, New York, United States of America and Dominica; 3 Massachusetts Institute of Technology, Cambridge, Massachusetts, United States of America; 4 Cummings Electronics Labs, Inc., Kingston, Massachusetts, United States of America; 5 Google Inc., Mountain View, California, United States of America; 6 Department of Marine Technologies, University of Haifa, Haifa, Israel; 7 Faculty of Electrical and Computing Engineering, University of Zagreb, Zagreb, Croatia; 8 Department of Natural Sciences, Baruch College and The Graduate Center PhD Program in Biology, City University of New York, New York, New York, United States of America; 9 Carleton University, Department of Biology, Ottawa, Ontario, Canada; MARE – Marine and Environmental Sciences Centre, PORTUGAL

## Abstract

Over the past decade, bioacoustics associated with diverse marine life has become the focus of increasing research. While fixed acoustic devices play important roles in characterizing localized soundscapes, animal-worn devices that record audio alongside physiological metrics provide richer portals to understanding cetacean communication and characterizing sounds in their environment. To facilitate scaling the collection of such multimodal datasets for deep learning applications and to encourage rapid prototyping for new recording capabilities, we present an open-source non-invasive bio-logger that can be deployed on marine animals to record high-quality audio synchronized with an extensible suite of behavioral and environmental sensors. The current implementation is tailored to investigating sperm whale communication and biology. It features four suction cups, three high-bandwidth synchronized hydrophones for audio analysis including directionality, GPS logging and transmission, and sensors for pressure, motion, orientation, temperature, and light. Its hardware and software are both open-source, with designs, fabrication details, and code available online. Lab-based experiments characterize and validate performance including shear adhesion forces, withstanding pressures equivalent to 560 m depths, battery life up to 16.8 hours, audio sensitivity of –205 dB re FS/μPa with a 96 dB dynamic range, multi-threaded data acquisition, drone-based deployments, and GPS-based recoveries. Field experiments record sperm whale vocalizations and behaviors spanning 10 deployments, 44 hours of recording, 20 dives, and up to 967 m depths. Altogether, this platform aims to advance the understanding of marine animal biology and communication within the rapidly evolving and intersecting areas of robotics, bioacoustics, and machine learning.

## 1 Introduction

The combination of bioacoustics data and modern deep learning techniques has vast potential for better understanding marine and terrestrial behaviors and environments, and has been described as opening up new avenues for inquiry [1–3]. In particular, while human knowledge of terrestrial organisms extends back thousands of years, deep marine environments and deep-diving marine mammals have only recently become accessible to human study due to technological advances that enabled a portal into these ecosystems [4]. Sound is a paramount sensory cue for marine mammals [5] that travels far through the ocean, and they have therefore evolved complex apparatuses to produce and detect sounds ranging from infrasonic to ultrasonic.

Leveraging technological advances to collect and curate large acoustic and behavioral datasets focused on marine animals could fuel data-driven approaches to scientific discovery. Recent advances in compute power and machine learning could then be applied to help extract insights into how marine animals interact with each other and how human activity affects these interactions. These, in turn, could inform sustainable paths forward for developing solutions that monitor, protect, and manage marine ecosystems.

To move towards this vision, there remain underyling challenges to streamline and scale data collection and curation. Continuous recording devices and discrete deployments can help increase the data captured about marine animal communication, but such devices will need to be designed specifically for interdisciplinary field use and tuned to machine learning pipelines. For example, since recorded vocalizations are not typically annotated, any synchronous behavioral information will be valuable for providing models with contextual grounding. Multimodal recording platforms that can be rapidly modified, fabricated, and deployed across research and field communities will thus be critical for expanding our foundation of data from which to extract insights. Integrating these multimodal platforms, such as on-animal bio-loggers capable of continuous acoustic recording, within sensor networks could help pave the way to applying modern data-driven approaches; techniques such as large language models and unsupervised deep representation learning hold potential to uncover structure in non-human communication and to yield predictive or translational capabilities, but they rely on datasets that are orders of magnitude greater than what has so far been gathered in the domain of marine animals [2]. Scaling data collection and synchronizing it with behavioral information could thus help move towards these possibilities, and these in turn may provide insights into properties of the vocalizations that help guide future versions of the recording devices.

Sensorized tags, or bio-loggers, are increasingly used to drive multimodal data collection in the ocean [6–11]. Such bio-loggers attach directly to marine wildlife and continuously record sensor data about both the tagged animal and their surrounding environment, offering a unique vantage point for gathering data. They do not disturb natural interactions, and they require only minimal human supervision.

However, there are numerous challenges to designing such bio-loggers and to using them at scale. Their hardware must withstand harsh conditions including corrosive saltwater and high pressures, and must also be compact and unobtrusive for the animals that they monitor. Their software must reliably stream from multiple sensors and operate without external connectivity for long periods of time, despite limited onboard computational resources [9]. High-quality audio is also critical for studying conversations between animals and for allowing machine learning pipelines to uncover structure in both time and frequency domains; bio-loggers should thus be sensitive across a wide range of amplitudes to record louder vocalizations from the focal animal as well as softer vocalizations from distant animals. Recording from multiple hydrophones that are spatially separated also aims to enable the inference of audio angle of arrival and thus facilitate source separation; this can be highly valuable for parsing social interactions by facilitating speaker separation across time or within overlapping vocalizations. However, enabling such techniques requires precise synchronization between multiple audio channels at recording time.

In addition to the technical challenges of sensing and mechanical infrastructure, there are important adoption and logistical considerations when aiming to scale the quantity of deployed bio-loggers. Perhaps most importantly, the bio-logger must be readily usable and valuable to multiple research groups around the globe to encourage more widespread usage of these devices. In addition, scalability will also require automated deployment solutions that reduce the manual labor needed to find target animals, attach bio-loggers, and recover bio-loggers.

**Fig 1:**
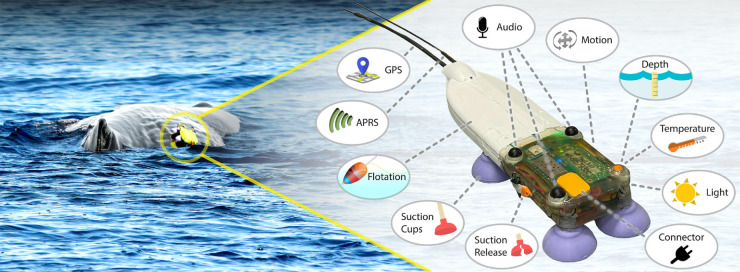
The CETI Bio-Logger on a wild sperm whale (left) and an overview of its main features (right). This bio-logger is used by Project CETI to study the communication of sperm whales in the Eastern Caribbean off the coast of the island of Dominica. It is intended to be deployed on sperm whales in a non-invasive way (with suction cups) and record high-quality audio.

Taking a step towards addressing these challenges, we present the CETI Bio-Logger shown in Fig 1. It represents an open-source and extensible multimodal recording platform. Design, fabrication, and software information are available at [12] and in the supplementary materials; by making all aspects of the bio-logger open-source, we aim to encourage multiple research groups to fabricate and deploy devices in a wider variety of locations and across more continuous timescales. Furthermore, designing bio-logger components in a way that is compatible with hobbyist-level tools to implement new sensors or recording behaviors aims to make the platform valuable for a wider variety of research endeavors. The software can be rapidly adjusted to accommodate additional sensors, and can adjust recording profiles in part based on real-time streaming data. Furthermore, scalable deployment and retrieval methods that build upon related work in such areas have been tested with this platform to move towards large-scale automated operations.

The bio-logger currently presented has been tailored to the study of sperm whale (*Physeter macrocephalus*) communication as part of Project CETI [13]. Sperm whales use a variety of vocalizations, including a communication system based on clicks and an echolocation system for navigating and locating prey. The CETI Bio-Logger enables data-driven analysis of these vocalizations by collecting high-quality audio from three hydrophones, in addition to synchronized behavioral information from pressure, motion, temperature, and light sensors.

This paper describes the CETI Bio-Logger platform and development process. In particular, it discusses the following primary features and contributions:

- Open-source hardware and software to encourage adoption across research teams [12].
- Extensibility to additional sensors and to additional marine species.
- Four high-bandwidth synchronized hydrophone channels that facilitate inference of audio source angle of arrival, three of which are populated in the current implementation, with gains adjustable at fabrication.
- A modular software architecture for adaptive multimodal recording, centered around a popular hobbyist platform to support rapidly prototyping new capabilities.
- A drone-based deployment procedure for less obtrusive tagging and to facilitate future automated operations.
- A GPS-based recovery system to reduce manual effort and facilitate automation.
- Results from lab tests, open-water tests, and deployments on wild sperm whales.

## 2 Related work

In recent years, there have been numerous investigations to develop underwater bio-loggers and to use their data to learn about marine environments. These efforts have led to advances for bio-logger hardware, deployment processes, and data analysis pipelines.

### 2.1 Bio-loggers

Bio-loggers have enabled breakthrough marine biology research by enabling continuous underwater monitoring of a tagged animal, studying both individual and group behaviors [14, 15]. Some of the most common bio-loggers focused on acoustics and movements for marine mammal research include the DTAG [16], the CATS tag [17], and the Acousonde [18]. They typically use suction cups to adhere to the animal, and record for a few hours up to a few days depending on the configuration. The bio-loggers often feature a subset of sensors that may include one or two hydrophones for audio recording, pressure sensors, temperature sensors, and inertial measurement units (IMUs) for motion and orientation.

The presented CETI Bio-Logger builds on these foundations to develop, test, and release an open-source bio-logger that, in this instance, was tailored to studying sperm whale communication but that can be adapted to additional use cases. It aims to provide a platform that can be extended in both hardware and software. Detailed hardware documentation encourages reproducibility, and a modular software architecture centers around an embedded computer with widespread community support to facilitate rapid iteration and customizability. The sensor suite focuses on contextualized high-quality audio, including the potential for estimating audio arrival directions, and can be extended to include additional sensors ranging from behavioral metrics to water quality for wildlife and environmental monitoring.

### 2.2 Deployment and recovery

For sperm whales, bio-loggers are typically deployed in the wild by researchers locating a target animal, approaching with a boat to within a few meters, and using a long pole to place the bio-logger onto the animal. This allows for manual placement and alignment of the bio-logger, and can be relatively unobtrusive depending on the required boat distance and the species. However, this requires substantial human time, effort, and training to complete successfully. This level of labor limits the possible scale of data collection efforts.

Similarly, recovery of the bio-loggers after data collection can be very resource intensive; common approaches include very high frequency (VHF) beacons on the bio-loggers that can be localized by an operator listening with specialized antennas and using this information to search for the bio-logger in a boat. Efforts to reduce labor during the recovery process include automating the directional estimation of VHF pings using antenna arrays [19], and using ARGOS satellite message Doppler shift to perform localization [20].

The presented work aims to move towards automating these processes to scale operations and increase the size of achievable datasets. On the deployment side, the CETI Bio-Logger can be deployed by traditional pole methods but has also been reliably deployed using manually piloted drones. Using drones expands the range of possible marine species to monitor by having added speed and maneuverability, as well as offering more minimally invasive deployments by not having a tagging boat in close proximity to the animals [21]. On the recovery side, the CETI Bio-Logger supports two protocols that have been leveraged in the biologging community. It broadcasts VHF pings which can generally be received up to 6 NM away via a directional antenna (Yagi) at sea level. GPS coordinates are also broadcast in VHF data packets to ease recovery efforts and facilitate future automated recovery solutions.

### 2.3 Uses of bio-logger data

Bio-loggers [7, 22] provide valuable datasets that can be used to better understand marine animals and ecosystems [11]. They have been used to study a wide variety of animals including sharks [23], dolphins [24], penguins [25], whales [26, 27], and many others [28]. For cetacean research, these bio-loggers have yielded insights into behavior [29], vocalizations [30], and animal health [31].

The current implementation of the CETI Bio-Logger platform is specifically focused on listening to and decoding sperm whale language [2]. This has informed the specifications for the current bio-logger, including high-quality audio from multiple hydrophones and the suite of contextual behavioral sensors selected. The inclusion of multiple hydrophones enables audio source direction estimation [32], which can be important for studying audio scenes that include multiple whales socializing. This work applies a preliminary pipeline based on times of arrival to experimentally validate the potential for this capability.

These efforts aim to move towards supporting data-driven approaches to understand the behavior of sperm whales or other marine species, along with anthropogenic impacts on that behavior. Studies have demonstrated such potential, using data to explore the impact of shipping noises [33], pollution [34], and climate change [35]. They have also laid the foundation for machine learning pipelines that are capable of offering deeper insights into communication systems and social interactions [36–40].

## 3 Overview of goals and contributions

The immediate goal of studying sperm whale language and behavior, together with the longer-term goal of facilitating more widespread bio-logger-based research, has guided the hardware and software specifications for the presented bio-logger.

### 3.1 Extensible and open framework

While the CETI Bio-Logger has been designed and used for sperm whales, facilitating widespread use of these tools across the research community will help scale data collection, encourage broader interdisciplinary collaborations, and lead to new discoveries. Towards this end, the project has focused on developing modular hardware and software that can be adapted to various sensor suites, creating open-source software that can operate on commodity computational resources, and releasing fabrication specifications. These efforts aim to move towards democratizing the fabrication and modification of recording devices capable of monitoring deep-diving marine wildlife, enabling more research groups to access such technology and tailor it to their purposes using hobbyist-level programming and prototyping.

### 3.2 Audio recording specifications

A primary function of the CETI Bio-Logger is to record high-quality audio from a single sperm whale or groups of interacting whales. In particular, temporal and spectral features of loud vocalizations of the focal whale should be captured without distortion to provide automated detection pipelines with as much information as possible and to allow learning pipelines to uncover structure in both the time and frequency domains. This implies that avoiding clipping from the focal whale is a paramount concern. At the same time, capturing interactions between animals implies that the system should also detect vocalizations from non-focal whales that are as distant as possible. Trading off these goals of detecting both loud and soft vocalizations, the current implementation has an end-to-end sensitivity of –205 dB re FS/μPa with a bitdepth of 16 bits. This enables the CETI Bio-Logger to capture levels from 120 to 205 dB re 1 μPa (peak). Given that sperm whale vocalizations of the focal whale at the bio-logger location are typically 180 dB re 1 μPa (peak) [41] and that off-axis vocalizations from an even closer whale 1 m from the bio-logger can be over 190 dB re 1 μPa (peak), these settings achieve the goal of avoiding clipping from nearby vocalizations. It also enables detecting distant vocalizations up to approximately 2.5 km away.

Regarding frequency content, the bio-logger should faithfully record between 1 kHz and 40 kHz to comfortably cover the frequency range of both communication and echolocation clicks which contain energy  to over 20 kHz [42, 43]. This implies that a sampling rate of at least 80 kHz is required based on the Nyquist rate; a standard audio rate of 96 kHz was selected to meet this criterion.

An important feature for post-processing capabilities, robustness, and extensibility is also supporting multiple synchronized audio channels. This can provide the following key benefits:

- Standard techniques such as analyzing differences in arrival times between spatially separated hydrophones can infer the direction of an audio source relative to the bio-logger; this can be crucial for separating vocalizations from interacting animals that may overlap in time. Doing so requires a high degree of synchronization across the audio channels, which can be difficult to design and implement. In particular, to provide as much angular resolution as possible, the recorded audio streams should be synchronized to within 1 audio sample.
- Multiple channels can be configured with different sensitivies to provide additional acoustic information. For example, they could be configured with varying gains to better capture near or distant vocalizations, or could be used to better characterize ambient noise data.
- Multiple channels can provide redundancy and thus robustness in the case of damage or runtime errors during deployments in extreme environmental conditions.

To address these goals, the CETI Bio-Logger features four audio channels that are synchronized to within 1 sample. Three of these channels are currently populated with hydrophones, and the final channel is available for future expansion. All channels currently use the same recording configuration, but this can be adjusted as desired to address varying research goals.

### 3.3 Multimodal data types

Additional data streams can help contextualize the recorded audio to better understand both communication and behavior.

Diving is a primary activity for the whales, and relates to aspects such as feeding, sleeping, and socializing. Recording dive profiles via pressure sensors is therefore a crucial data stream [14, 44].

In addition, instantaneous acceleration, rotation, and heading provide valuable insights into whale motion. Analyses focused on short timescales, including instantaneous acceleration or rotation, may provide useful information about fast motions such as feeding lunges. Analyses focused on longer timescales, such as average whale orientation over minutes or hours, may provide useful information about activities such as sleeping vertically [45].

Finally, information about the surrounding environment provides context for such activities; relevant characteristics may include temperature and light levels.

### 3.4 Global synchronization for dataset curation

While bio-loggers can provide rich information about the tagged individual, synchronizing multiple independent sensing platforms can yield more complete descriptions of interactions and more contextual information. Such information can be critical for machine learning pipelines that aim to decode communication. Towards this end, the bio-logger should timestamp samples within each data stream using a synchronized world clock. This can facilitate alignment with platforms such as bio-loggers on other animals, aerial or underwater drones, passive listening stations, or other robotic devices. The resolution of synchronization needed will depend on the application. For the timescales of behaviors and vocalizations currently being considered, alignment on the order of one second can be sufficient and achievable via standard internet- or GPS-based timing methods. Note that once a time-based synchronization is established between two sensors, the data itself can often be used to refine that alignment if needed.

### 3.5 Robustness and operational duration

The bio-loggers must be robust to harsh conditions, and adhere securely to the animal to record data for sufficient durations to span all notable behavioral cycles. To be deployed on a whale (by pole or drone tagging) and to adhere long enough, the bio-logger must weight less than 1 kg and its drag forces must be lower than the retention shear force provided by the suction cups.

The target operational duration is 12-24 hours; this considers behavioral cycles including diving and sleeping, as well as deployment and recovery logistics. Limiting the travel distance of the whale facilitates successful bio-logger recovery, since recoveries in open ocean far from land can increase risks for the recovery team. Note that sperm whales exhibit behavioral cycles longer than the targeted operational duration, such as diurnal cycles or even annual cycles, but the current bio-loggers aim to sample notable portions of the behavioral cycles and capture key discrete events. For example, typical dive cycles are under 1 hour and sleep cycles are on the order of 15 minutes. Deploying multiple bio-loggers simultaneously or over time could increase behavioral coverage in the future.

To ensure desired limits on adhesion duration, the bio-logger should include the ability to automatically release the adhesion system based on pre-programmed criteria.

Given that sperm whales typically hunt at depths ranging from 400 to 1 200 m, [46], the bio-logger needs to withstand associated pressures for approximately 45 minutes per dive-surface cycle. Finally, the bio-logger should resist corrosion and water ingress despite days of submersion in saltwater.

### 3.6 Deployment and recovery operations

Just as important as developing capable hardware and software is ensuring the bio-logger can be readily used in the field by users with a wide variety of expertise. This requires hardware and software initialization procedures that are reliable and automated. For example, a user interface with simple visuals indicating bio-logger state is essential (e.g., powered on, ready to deploy, and any errors). The bio-logger should have accessible calibration procedures for sensors such as IMUs that need to be calibrated in the field, and largely automated procedures for downloading data from the bio-logger and adding it to curated datasets (e.g., cloud-based datasets).

In addition, curating datasets that support deep learning pipelines will require scaling operations to collect data more continuously from more animals. This requires rapid and automated methods to deploy bio-loggers, for example using drones. The chosen deployment methods must be minimally obtrusive to the animals, especially as operations scale to more frequent deployments. And finally, recovery methods are needed to reduce manual effort and to facilitate future automation including drone-based recovery.

## 4 Bio-logger components and fabrication

### 4.1 Bio-logger body and assembly

The main components of the CETI Bio-Logger are illustrated in S3 Fig, and its overall dimensions are shown in S1 Fig. It is organized such that all (dense) electronic components are on the anterior portion and all flotation is posterior. This buoyancy distribution creates a stable orientation when the bio-logger is at the surface that ensures the communication components housed in the flotation section are out of the water; these include an APRS antenna and a VHF fish tracker (F1840B, Advanced Telemetry Systems, Isanti, MN, USA).

Syntactic foam (MZ-22, Engineered Syntactic Systems, Attleboro, MA, USA) is used for flotation due to its low density, low water absorption, and robustness to high hydrostatic pressure. It can be machined to desired specifications [47], is widely used in oceanographic applications [48], and is rated up to 1 000 m.

Making the bio-logger robust to greater depths involves tradeoffs for component selection (such as increased size and cost), and devices rated beyond 1 000 m may be regulated by various jurisdictions (e.g., the United States Department of Commerce or Department of Defense). Nevertheless, note that testing procedures should incorporate generally accepted safety factors for the expected dive conditions for particulary fragile components, especially considering potentially dangerous implications such as battery failures, and consider incorporating safety mechanisms such as releasing the bio-logger if the depth is within a factor of safety of the rated depth (e.g., less than 1 000 m for the CETI Bio-Logger).

The part of the bio-logger body that houses the electronics is filled with epoxy for waterproofing and to withstand the high hydrostatic pressures associated with deep diving; the injection process is shown in S1 Appendix - Sect 2.1.

### 4.2 Hydrophones

The CETI Bio-Logger supports up to four hydrophones to record acoustic data, three of which are currently populated. Each hydrophone consists of a piezoelectric ceramic sphere (10 mm in diameter) surrounded by acoustically transparent rubber for protection and index matching; S1 Appendix - Sect 2.2 summarizes the manufacturing process.

### 4.3 Suction cups and Burnwire release system

During data acquisition, the bio-logger adheres to the animal using four compliant suction cups (silicone rubber with a shore hardness of 45A) whose design is inspired by existing work [17]. These suction cups, along with the deployment procedures, aim to limit impact on the animal; they do not penetrate the skin, and as such pose little to no negative impact on the study animal [49] while enabling sufficiently long recording sessions to capture key behavioral cycles, e.g., from 12 to 24 hours.

The suction cups are manufactured with injection-molded silicon rubber. The fabrication process is based on a previously established technique [50, 51] and is described in S1 Appendix - Sect 2.3. A threaded component is embedded in the silicone so the cups can be screwed into the bio-logger for quick replacement.

As with other marine mammal bio-loggers, suction can be programmatically released via a burnwire system. This allows the bio-logger to be recovered more easily at a given time, with the drawback of reducing the average whale adhesion time. To fulfill this purpose, a through-hole in the suction cup connects it to pneumatic tubing that is kinked to prevent fluid flow during normal operation. A nickel-chrome wire measuring 0.3mm×0.1mm (WireOptim, Bradenton, FL, USA) holds these tubes in place, but applying a voltage across this wire while underwater causes corrosion that burns the wire and allows the tubing to straighten and release suction. A schematic of the release system is described in S1 Appendix - Sect 4. This mechanism thus allows the bio-logger software to detach the bio-logger from the animal in response to specified conditions such as low battery voltages or user-specified recording durations (e.g., release before nighttime to ease recovery). The programmed release could also be used in combination with GPS localization to trigger release when the whale leaves a predefined region (e.g., geofencing). When the burnwire is engaged, it uses approximately 120 mA of current and takes on average two to five minutes to release depending on the environmental conditions such as the water temperature and salinity.

### 4.4 Sensors and general electronics

**Fig 2:**
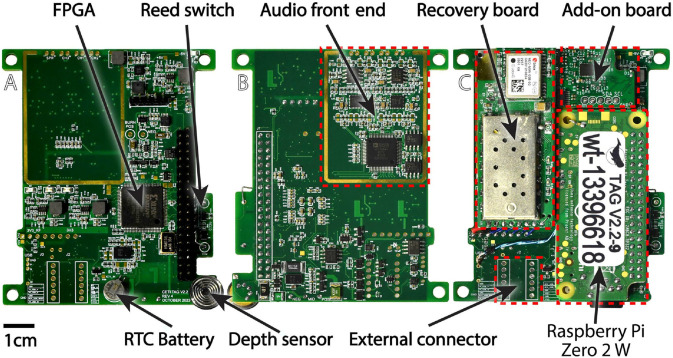
A six-layer PCB hosts all sensing, data acquisition, and computational electronics. A: Top view before populating the Raspberry Pi and the recovery daughterboard. B: Bottom view. C: Fully populated top view.

**Fig 3:**
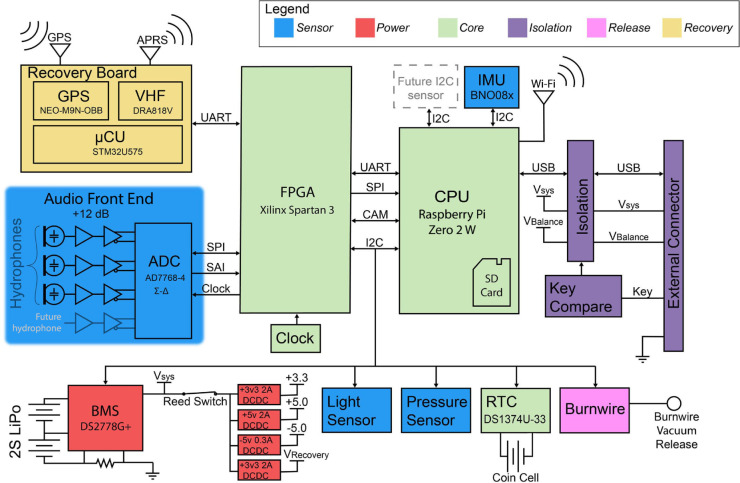
System block diagram of CETI Bio-Logger electronics.

The CETI Bio-Logger consists of a single-board computer (Raspberry Pi Zero 2 W, Raspberry Pi Holdings, Cambridge, England, UK) mounted on a custom six-layer PCB as shown in Fig 2. This PCB features a four-channel digital audio recording system along with a battery management system (BMS), real-time clock (RTC) to provide second-accurate timing across reboots when network communication is not available, a pressure sensor, temperature sensors, an IMU, an ambient light sensor, the burnwire release system, and a field-programmable gate array (FPGA). Fig 3 provides a system-level view of how these various parts are connected and how they communicate with each other.

The board also features two expansion headers that can be used to implement additional functionality via industry-standard serial links. For example, the current version of the CETI Bio-Logger uses one of the expansion headers for a VHF-based recovery system.

The FPGA allows data lines to be easily redirected to the Pi, enables high-speed audio signal preprocessing, and provides a method to gracefully power down the system when battery levels are critical.

#### 4.4.1 Sensing capabilities.

Data from a suite of sensors provides additional behavioral and environmental context for the bio-logger’s recorded audio.

**Pressure and Depth:** The pressure transducer (4LD, Keller, Switzerland) is an oil-filled MEMs device which can measure pressures from 0 to 20 MPa (200 bar) with an accuracy of ±300Pa(±0.003bar) [52]. The depth of the bio-logger can be calculated using these pressure measurements and the approximate bio-logger latitude [53].

**Water Temperature:** The above pressure sensor also measures ambient temperature with an accuracy of $\pm 2^{\circ }\;\text{C}$.

**Motion and Orientation:** The IMU (BNO086, CEVA Technologies, Rockville, MD, USA) [54] includes an accelerometer, gyroscope, and magnetometer. It also fuses these readings to estimate the orientation of the device.

**Ambient Light:** An ambient light sensor (LTR-329ALS-01, Lite-On Inc., Taipei, Taiwan) captures both visible and infrared light, which provides information about when the whale surfaces and when the bio-logger is occluded by other whales or by changes in orientation.

#### 4.4.2 Waterproofing and robustness considerations.

Given the harsh operating environment, several additional features and redundancies have been incorporated into the CETI Bio-Logger’s electrical design. Wi-Fi and Ethernet via an isolated USB connection provide redundant methods of connecting to the bio-logger for programming, reconfiguring the device, updating firmware, and offloading data. Since the current bio-logger implementation records approximately 334 MB of data per hour, fast and redundant methods of downloading the data is important for smoothing field operations and reducing bio-logger downtime. The bio-logger features external connections for USB and battery charging. During normal operation, these are electrically isolated from the main board to reduce the effects of corrosion due to electrolysis. When a pre-set voltage is applied to the external connector via a custom dongle, a comparator on the bio-logger switches off this isolation and allows the tag to be charged or connected to Ethernet via USB. The burnwire release system features electrical isolation for when the release is not activated, and a 200 mA resettable fuse to limit the power drawn from the batteries when the bio-logger is releasing. During releasing, the burnwire system consumes approximately 120 mA.

### 4.5 Audio recording system design

The CETI Bio-Logger audio acquisition electronics and software are designed to provide up to four channels of simultaneously sampled 24-bit audio data. In the current configuration deployed for sperm whale recordings, three channels are captured at 96 kilo-samples per second (kSPS) and stored with 16 bits of resolution. The hydrophones are arranged in an “L” shape to enable direction-finding via phase or correlation analysis.

#### 4.5.1 Sensitivity and dynamic range.

Deciding the sensitivity and dynamic range of the CETI Bio-Logger requires tradeoffs among the nature of the signal of interest, system power consumption, analog bandwidth, amplifier selection, and data throughput. For the sperm whale application, detecting high-intensity signals from the tagged whale itself (i.e., the focal whale) without clipping is prioritized, and additionally detecting attenuated signals from distant whales is valuable for analyzing social interactions. In addition, an appropriate dynamic range could allow the bio-logger to record click waveforms from a distant whale with minimal distortion, equivalent to being received by the listening whale. The peak recorded sound pressure level (SPL) of the signal from the focal animal is a function of bio-logger placement but is routinely greater than 180 dB re 1 μPa (peak) [41]. The level of a vocalization at a 1 m distance is approximately 190 dB re 1 μPa (peak).

In the current configuration to record sperm whales, CETI Bio-Logger uses 16 bits of resolution with an end-to-end sensitivity of −205 dB re FS/μPa. This means that a 205 dB re 1 μPa (peak) sperm whale vocalization will generate a full-scale positive peak output of 32 767 counts from the analog-to-digital converter (ADC). It can therefore successfully record a focal whale vocalization without clipping.

In addition to avoiding clipping of large signals, it is desirable that the noise floor of the recording system is limited by background noise of the underwater soundscape rather than electronic self-noise so that sounds from distant whales and other sources can be captured. Ambient ocean noise levels decrease with frequency and vary strongly with conditions. A quiet ocean exhibits noise levels below 30 dB re 1 μPa^2^/Hz at 10 kHz [55, 56]. This suggests that a dynamic range of over 140 dB is required to record ambient noise in the band of interest while at the same time avoiding clipping. In practice, this level of dynamic range is difficult to achieve and tradeoffs with respect to power consumption and the performance of available electronic components must be explored.

The RMS noise floor of the current implementation when measured in the laboratory is typically 110 dB re 1 μPa, which is close to the 16-bit quantization limit. Deployment data collected so far has exhibited RMS background noise ranging between 112 and 120 dB re 1 μPa with a low-pass frequency distribution concentrated below 500 Hz. This component of the signal can be attributed in part to flow noise that is dependent upon the location of the bio-logger on the whale’s body as well as the whale’s motion. These settings have been sufficient for recording high-quality sperm whale vocalizations, but increasing bit depth and gain adjustments have been explored to obtain an extension of the dynamic range even closer to the theoretical ambient limits and is the subject of continued work.

#### 4.5.2 Acquisition hardware and signal filtering.

Each channel of the analog front end presents a 374kΩ impedance to the hydrophone. The hydrophone has a nominal capacitance of 6.8 nF and forms a high-pass filter with the input resistance having a cutoff frequency of 63 Hz. This removes DC offsets and serves to attenuate low-frequency content associated with ocean ambient background noise, vehicle noise, and water flow noise.

A JFET op amp in a non-inverting configuration buffers the hydrophone and provides +12dB of gain and an active low-pass filter with a cutoff set at 100 kHz. This serves to prevent aliasing of out-of-band signal and noise. It should be noted that since the ADC is a sigma-delta architecture, anti-aliasing requirements are relaxed considerably [57], and a first-order filter provides sufficient rejection of out-of-band aliases in most cases.

The ADC used to digitize this filtered signal is the Analog Devices AD7768-4 [58], a four-channel sigma-delta converter that provides a rich set of programmable and flexible features that allows the designer to balance requirements for power, input bandwidth, and output data rate. Output data can be generated at up to 256 kSPS and the input section supports a bandwidth of 110 kHz with a dynamic range of 108 dB.

The CETI Bio-Logger uses a CMOS 98.304 MHz crystal oscillator with an accuracy of 25 ppm as the primary acquisition timebase for the audio digitizer. The output of the oscillator is frequency-divided by four to create the ADC master clock at 24.576 MHz. To establish the Output Data Rate (ODR) of 96 kSPS, the master clock is further divided by eight internal to the ADC to generate the 3.072 MHz sigma-delta modulator clock. The output of each modulator is decimated by a factor of 32 to achieve the final output rate of 96 kSPS per channel. It should be noted that the ADC provides considerable flexibility with respect to clocking and sampling and can be adjusted via software changes or a configuration file for various applications. Notably, power consumption can be reduced considerably by employing a lower sampling rate.

The ADC also provides built-in digital filters for each channel. One of two filter types may be selected at run time. For the sperm whale application, the bio-logger currently applies wideband filters which have a nominal cutoff frequency of 0.4×ODR, which is close to the content that is recoverable based on the Nyquist rate; this filter has a cutoff of 38.4 kHz given the current ODR of 96 kSPS. Additional filtering may be applied as a post-processing step to further study the the spectral components of the signal.

#### 4.5.3 Sampling and compression.

The four channels of 24-bit data are transferred continuously to the bio-logger’s FPGA where each channel is truncated to 16 bits and then populates a FIFO buffer. Currently, data from one channel is not in use and is discarded. Note that the extra bits of ADC resolution and the extra channel are included for future extensibility, such as differing ambient noise floor levels than the current application or enabling additional angle of arrival capabilities. The Raspberry Pi reads the FIFO buffer via SPI, losslessly compresses the audio, and saves the data to the bio-logger’s microSD card.

An advantage of incorporating a full onboard computer is the ability to leverage compression algorithms that may be too computationally intensive to run on smaller platforms. The current implementation uses the FLAC format. This uses a lossless compression algorithm, and experimental results from sperm whale deployments indicate that the size of the compressed audio is approximately 15% of the raw audio data size. This reduction in file size by approximately 85% substantially increases the feasible recording duration given a fixed memory capacity.

#### 4.5.4 Multi-channel synchronization.

Regarding synchronicity between the multiple audio channels to enable post-processing (e.g., audio source localization), the selected ADC features a simultaneous sampling architecture which results in excellent phase matching between channels. Once the sample is taken, there is no additional error introduced in the digital pipeline; this results in a synchronization of less than one sample across all channels. Note that the exception would be if an overflow occurs due to a rare processing slowdown within the Raspberry Pi; the current software automatically detects such cases and initiates a reset of the audio recording pipeline.

#### 4.5.5 Extensibility.

The above audio acquisition hardware supports a range of configurations that can be specified via software, so research teams can modify the behavior to address varied use cases. Between one and four channels can be used with either 16 or 24 bits of resolution, and the sampling rate can be set up to 192 kHz. The current configuration of recording three 16-bit channels at 96 kHz achieves the target specifications for sperm whale recordings while also prioritizing data storage scalability.

Within the space of possible configurations, the current bottleneck is the 15 MHz SPI communication link between the FPGA and the Raspberry Pi; this limits the amount of audio data that can be captured before hardware changes would be required. Notable configurations that have been successfully tested at the frontier of this limitation include the following: two 24-bit channels sampled at 192 kHz, three 16-bit channels sampled at 192 kHz, three 24-bit channels sampled at 96 kHz, and four 16-bit or 24-bit channels sampled at 96 kHz.

## 5 Onboard software architecture

The onboard computational capabilities and software architecture were designed to enable synchronous multimodal streaming of audio and behavioral sensors, encourage rapid prototyping for diverse research goals, ease usability during field deployments, and facilitate the curation of a large-scale dataset of both bio-logger data and external devices such as drones or gliders. Challenges include limited computational resources of embedded devices, varied sampling rates across sensors, operation without human supervision, uncertain internet connectivity during deployments, no wireless connectivity underwater, possible intermittent power losses, and possible data corruption via water damage.

To achieve the desired capabilities and address the above challenges, the CETI Bio-Logger features an onboard computer with a widely supported operating system, programmable hardware connections to many of its ports, a modular multi-threaded software architecture that leverages multiple CPU cores for reliable streaming, a multi-tier software approach to ensure reliability, an adaptive state machine to automatically adjust recording behavior based on deployment milestones, and human-readable output formats that are also amenable to large dataset integrations. All software is open-source and available online [12]. Note that the current platform emphasizes adaptability, rapid prototyping, and community accessibility; once a design and capabilities are finalized for a particular use case, they could potentially be migrated to a design based on a low-power microcontroller if runtime and size are key priorities.

### 5.1 Embedded computational capabilities

Computation on the CETI Bio-Logger revolves around a Raspberry Pi Zero 2 W. This is a widely used hobbyist platform with a large basis of community support, which helps make programming or modifying the bio-logger behavior accessible to varied research groups. It includes a 1 GHz quad-core CPU, 512 MB of RAM, and Wi-Fi capabilities for wireless programming and data transfer. The SD card capacity can be chosen based on the target application; currently, a 256 GB SD card is used to expand the data recording duration beyond what is expected by battery life and adhesion limitations. The Pi runs a Linux operating system, and the main code for the bio-logger is implemented in the C programming language.

In addition, an FPGA (XC3S200A-5VQG100C, Xilinx, San Jose, CA, USA) is used to route many of the signals from sensors to the Raspberry Pi. This adds an additional computational resource for high-speed signal preprocessing, and allows many physical connections to be updated after fabricating the electronics and sealing the bio-logger.

### 5.2 Embedded software architecture

**Fig 4:**
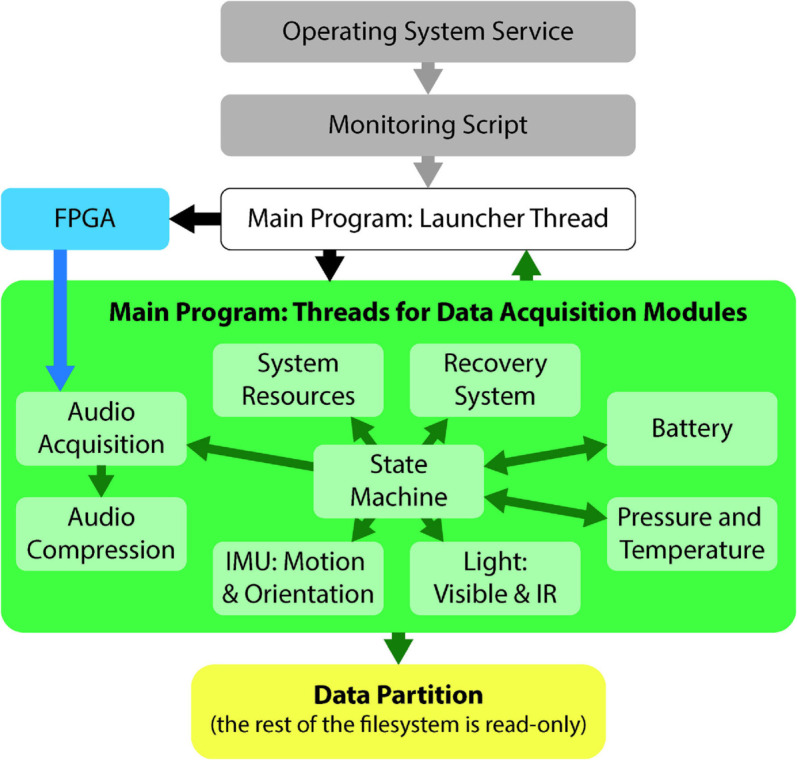
The software is designed in a modular architecture. Central management threads and a dedicated data partition ensure reliability. Individual data acquisition threads facilitate extensions to new sensors, accommodate varying sampling rates, and leverage multiple CPU cores.

The software architecture aims to provide reliable synchronous sampling of multiple sensors, adapt behavior throughout a deployment, and be robust in harsh conditions. It is arranged in a modular framework as shown in Fig 4.

#### 5.2.1 Multimodal data acquisition.

The software consists of independent data acquisition modules with common interface functions, which are overseen by a central manager. This modularity allows each sensor to operate at a sampling rate that is most appropriate to its modality, and aims to facilitate swapping sensors or capabilities in the future with reduced coding overhead. The central manager uses common interface functions for each module to start and stop the threads at appropriate times, ensure that they remain operational, and monitor system resources.

To best utilize the available computational resources, each data acquisition module operates in its own processing thread. The operating system can choose processing cores for these threads based on real-time demands, and modules with high sampling rates and time-sensitive demands can also be assigned dedicated cores. For example, the audio acquisition thread is assigned to an isolated core and no other threads or operating system tasks are allowed to use that core.

To prepare recorded data for integration within larger datasets from additional recording devices, each data acquisition module saves its data to a dedicated output file with timestamps for each acquired sample in a world clock reference frame. Timestamps are relative to a global epoch as provided by the system clock; this clock is synchronized to global time servers via Network Time Protocol (NTP) if internet access is available upon bio-logger startup. At the same time, the onboard RTC is also updated; this clock can then be used later to synchronize the system clock if no internet access is available upon bio-logger startup. This RTC operates independently of the main bio-logger infrastructure, and has a backup battery to continue operating across bio-logger power cycles. This process helps ensure that all samples are timestamped in a common reference frame that is also shared by external recording devices ranging from drones to passive buoys.

#### 5.2.2 Adaptive recording behavior.

**Fig 5:**
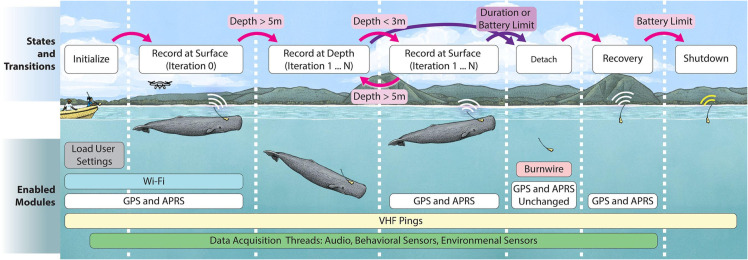
A state machine dynamically adapts the bio-logger’s recording behavior throughout a deployment. This includes initialization of systems such as real-time clocks, enabling or disabling wireless capabilities, launching or stopping data acquisition threads, releasing suction, and shutting down. Illustrations are based on graphics by Alex Boersma.

The central management system features a state machine that adjusts the recording behavior based on inferred milestones throughout the deployment. The states and transition logic are visualized in Fig 5, and key states are summarized below.

**Initialization:** The program loads user-specified parameters, initializes clocks, calls startup procedures for data acquisition modules, and launches data acquisition threads.

**Recording at the surface:** When the pressure sensor indicates that the current depth is less than 3 m, all data acquisition modules actively record and the recovery system is enabled. GPS information is acquired once per minute (when available), and location information is periodically transmitted. However, Wi-Fi remains disabled after a first dive since it is unlikely that an operator would be within range and attempting to access the bio-logger. Note that during field deployments, the effectiveness of GPS logging while the bio-logger was on a whale was intermittent. This is due to the GPS antenna, whose effectiveness degrades when wet and in a horizontal position along the whale’s body. This is being addressed in newer versions of the bio-logger through the exploration of alternative antenna designs.

**Recording during dives:** When the pressure sensor indicates that the current depth is greater than 5 m, all data acquisition modules actively record but all wireless communication capabilities are disabled to conserve battery power. These include the recovery system, GPS, and Wi-Fi.

**Recovery:** After a user-specified duration or after the battery level drops below 6.4 V, the burnwires are activated to ensure that the bio-logger detaches and floats to the surface. The start time for this countdown is recorded at the first detected dive. This time is saved to persistent storage, loaded from disk upon startup, and cleared after the burnwire activates; this allows operators to start the bio-logger at an arbitrarily long time before deployment for testing purposes or based on experimental conditions without risking premature detachment, and ensures that the proper detachment time is achieved even if the bio-logger has unexpected power cycles.

**Critical shutdown:** If the battery level drops below 6.2 V, all data acquisition modules are disabled to devote all remaining power to the recovery infrastructure. If the battery reaches a further critical threshold of 6.0 V, the bio-logger is powered off to protect the battery. At this point, the bio-logger can still be recovered via listening for pings from the VHF fish tracker with a directional antenna.

#### 5.2.3 Robustness and data integrity.

The central management system has a tiered approach to ensure that all data acquisition modules are reliably launched across reboots and remain running throughout deployments. A background script launches the main program and periodically checks its status, and operating system services ensure that this background script continues running.

To prevent corruption of the operating system in the case of unexpected power cycles, the filesystem is kept in a read-only state except for a dedicated data partition. Data acquisition threads for behavioral and environmental sensors write data to this partition in human-readable formats, and files are only kept open for the duration of writing a single sample. Data acquisition threads for audio use a dual-buffer approach to acquisition and writing, flush data every minute, and create a new audio file every five minutes. Together, these measures limit potential data corruption and facilitate data recovery in the event of unexpected power cycles.

## 6 Field operations: Usage, deployment, and recovery

### 6.1 Bio-logger usage

The hardware and software were co-designed to minimize time and technical expertise required to operate the bio-logger in challenging field operations. The bio-logger features a standard Linux operating system and thus its software can be updated wirelessly via Wi-Fi, but key parameters can also be adjusted without programming. Human-readable configuration files allow pre-deployment adjustments to settings such as pressure thresholds or the adhesion duration.

Before deployment, a hardware test script can be used that interactively prompts the user to test each subsystem and validates expected operation. This script creates a test file of the results which can be used to track the health of the bio-logger over time.

During a deployment, the operator simply removes an external magnet to power on the bio-logger. LEDs then indicate that the bio-logger has started successfully and is ready for deployment. Time permitting, an optional brief calibration for the IMU can be performed at any time by holding the bio-logger in perpendicular orientations and by rotating it around the three axes (but this is not required in order to use the data at the end of a mission).

After recovery, data can be offloaded wirelessly or via a wired connection. Scripts are provided to the operators that automatically copy data from the bio-logger, integrate it within the cloud-based CETI dataset, ensure successful data transfer, and clear the bio-logger to prepare it for a future deployment. These can be generalized for other researchers using local or cloud-based storage.

It is also important to have streamlined protocols for field teams to check that bio-loggers are functioning as expected. Scripts are provided that validate each subsystem, and these are incorporated into protocols to be run before each deployment. Additionally, over the lifespan of the bio-logger, testing and field data is regularly checked by the engineering and biology teams for irregular results or behavior. The inclusion of an onboard computer with wireless capabilities also supports the creation of a wireless dashboard to interactively visualize and stream bio-logger data for validation purposes, or automated system checks that upload results to a centralized database for tracking bio-logger health over time; these capabilities are being developed for future deployments.

This research has been conducted under the Dominica Fisheries Research Permit # LS 27-200-21 and Harvard IACUC Protocol ID # 21-02-379-1.

### 6.2 Deployment

**Fig 6:**
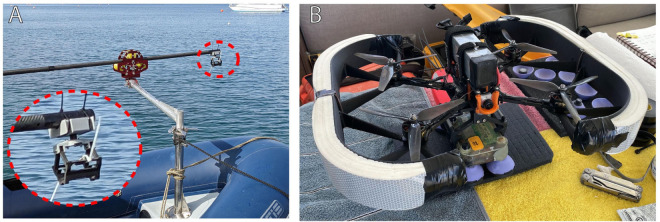
The bio-logger has been deployed using pole-based and drone-based methods. A: During pole-based deployments, a 7 m (21 ft) pole is fixed to a four degree-of-freedom arm mechanism that is mounted on the bow of a rigid inflatable boat (RIB) boat. B: During drone-based deployments, a modified racing drone holds the bio-logger with a bistable clamping mechanism.

The bio-logger has been deployed in two ways. The first method is the standard approach of using a long pole from a boat, as shown in Fig 6A. A human operator uses a bistable clamping mechanism to hold the bio-logger at the end of a long pole. The boat approaches close to the whale, at which point the operator places the bio-logger onto the whale’s dorsal side, avoiding the blowhole and dorsal fin. The deployment triggers the bistable clamp to passively release the bio-logger and allow it to remain adhered to the whale.

The second deployment method is drone-based tagging [59], as shown in Fig 6B. While our bio-logger is suitable for use in drone-based passive bio-logger drop systems [21], the presented deployments use an inexpensive and rapid method for deploying bio-loggers using small First Person View (FPV) racing drones, which are modified to be waterproof and buoyant. With the bio-logger secured in a similar bistable clamp as used in pole tagging, the drone descends towards a whale and performs a tap-and-go deployment on the dorsal side of the whale.

For both of these methods, strict animal tagging protocols are followed to respect the animal’s well-being (e.g., limiting the number of tagging attempts, identitifying all tagged whales for follow-up, documenting response levels, cessation of tagging based on identities of whales and behavioral state, and quantifying dive parameters after deployment–see [49, 60] for more information). Furthermore, Project CETI is active in the establishment of legal and ethical guidelines for nonhuman communication studies [61, 62].

### 6.3 Recovery

Given the high-quality audio recording system of the CETI Bio-Logger that generates approximately 300 MB of losslessly compressed data per hour, and the relatively short periods of time the bio-logger is above the surface while attached to a whale, data retrieval must occur after the bio-logger releases from the whale. This also aligns with the operation of previous sound-recording animal-borne bio-loggers. This need to reliably retrieve the bio-logger from the field motivated the implementation of two recovery methods for the CETI Bio-Logger.

The first method, which has been a standard of animal-worn technologies for decades, uses a standalone VHF beacon fish tracker (or “fish implant”) that is inserted in the rear flotation syntactic foam and does not require electrical integration with the main bio-logger. This device transmits a pulse every second, which can be received by a directional antenna to infer a relative heading.

The second method uses GPS and involves the development of an expansion board attached to the main bio-logger PCB. This board is capable of recording GPS information during the mission (when a signal is available), and transmitting GPS information to the field team for retrieval after the bio-logger has detached from the whale. Several land-to-land and land-to-space radio communication technologies were explored for position estimation and transmission, but ultimately the Automatic Packet Reporting System (APRS) was selected for use on the expansion board.

APRS is a well-established packet radio system intended for non-commercial use to track the position of moving objects. The protocol was established by the amateur radio community in 2000, and APRS networks now exist throughout the world [63]. The networks consist of VHF beacons that transmit frequency-shift keying (FSK) frequency modulating (FM) packets containing their position, “digipeaters” that receive these packets and repeat them to extend the range of the network, and “i-gates” (internet gateways) that receive the message and report them to the Automatic Packet Reporting System-Internet Service (APRS-IS) [64]. These messages can then be accessed anywhere in the world via APRS-IS or via various websites that map this position data in real time. APRS position data and direct messages can also be received locally by handheld radios supporting the protocol.

The current deployments have been conducted in Dominica, which makes APRS a particularly appealing choice. There is an existing APRS network on the island consisting of three stationary radio antennas acting as digipeaters and existing i-gates. The island’s mountainous geography also helps increase the range of this type of network. Beyond Dominica, the surrounding islands in the Caribbean also have an existing network of digipeaters [65].

## 7 Experimental results: Validation and characterization

### 7.1 Robustness and longevity

#### 7.1.1 Pressure cycling during simulated dives.

The CETI Bio-Logger has been tested using a high-pressure tank, reaching pressures that reflect diving conditions for over 100 rapid cycles. At the end of the experiment, a visual inspection is conducted to look for any crushing or deformation of the bio-logger. The bio-logger-measured pressure data is also compared with the pressure logger of the tank. S1 Appendix - Sect 6 provides additional information.

#### 7.1.2 Power usage and recording duration.

The CETI Bio-Logger is powered by two 5 000 mAh lithium ion batteries (PL-6767100-1C, AA Portable Power Corp, CA, USA) connected in series to produce a nominal voltage of 7.4 V. Total power required by the bio-logger when recording data is approximately 2.2 W, yielding an expected runtime of 16.8 hours. Power consumption can be tailored to some degree by enabling or disabling features, either dynamically via the autonomous state machine or via user specifications. S1 Appendix - S2 Table provides the approximate power usage of each of the bio-logger’s principal features.

#### 7.1.3 Adhesion characterization.

Suction cups were tested to evaluate the preload needed to fully compress the cups, as well as the displacement under shear loading. Using a robotic arm with a wrist-mounted force/torque sensor, and using parameters described in [51], cups were compressed onto a wet surface with a predetermined amount of preload force (e.g., to emulate deployment), which was then relaxed to generate an initial suction chamber. Cups were then sheared across a surface for a distance of 10 mm. This process is further detailed in S1 Appendix - Sect 3.

**Fig 7:**
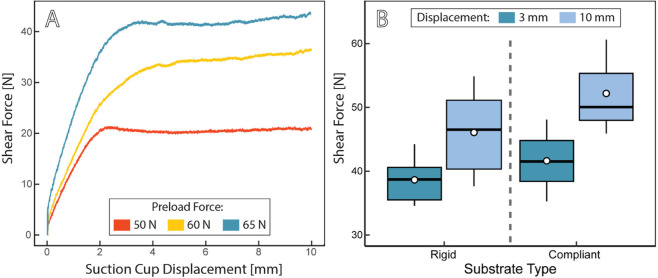
Suction cup performance was evaluated under shear conditions by compressing the cup to create adhesion, applying a shear force, and measuring the cup displacement. A: Shear resistance is much lower if the cup is not fully adhered before shear loading (using 50 N of preload force). B: The shear forces required to generate 3 mm or 10 mm displacements were comparable when testing on a rigid or compliant surface. Boxplots display median, upper and lower quartiles, inter quartile range (IQR), and outliers. White dots indicate means.

The initial compression preload was first evaluated on a smooth acrylic surface to determine how initial contact area influences shear resistance; results are shown in Fig 7A. When the cups were not fully compressed onto the surface before a chamber was generated, shear resistance was much lower. This is likely a result of the initial contact area created before the shear pull was initiated – higher preload yields more contact area, which yields more friction during shearing.

Using the higher preload configuration (65 N), additional tests were performed on two different substrates: smooth rigid acrylic and smooth compliant Smooth-Sil 945. Shear forces were recorded at two points: the 3 mm displacement, which represented the approximate point where the cup would begin sliding, and the 10 mm displacement at the end of the shear run. Results are shown in Fig 7B.

Evaluating the forces required to load and displace the suction cups is important for bio-logger deployment and the development of future suction cup designs. For example, if drone deployment does not generate sufficient force to fully engage the suction cups, shear loading can dislodge the bio-logger much more easily than if the cups were fully compressed. This could impact the design of the drone deployment mechanism. Additionally, softer or multi-material cup designs could be explored to reduce the amount of force needed to fully engage. Note that although the current suction cup has a rounded shape and a smooth contact surface, additional adhesion research has explored the potential of varying the suction cup shape [51] and rim structures [66] using similar fabrication techniques. Such designs are compatible with the CETI Bio-Logger and could be incorporated in the future.

### 7.2 Audio characterization

The sensitivity and frequency response of the bio-logger’s audio recording subsystem were characterized in a custom water tank. The water tank is 1.5 m deep × 2 m long × 2.4 m wide, which is large enough to characterize audio performance above 10 kHz [67].

The characterization experiments consisted of two phases. First, the response of the speaker was experimentally determined by using a reference hydrophone. Second, these results were used to emit pulses at equal amplitudes across a range of frequencies and measure the response of the CETI Bio-Logger audio subsystem. Fresh water at $23^{\circ }\text{C}\;\pm \;1^{\circ }\text{C}$ was used in the tank, resulting in a sound speed of 1 491.2 ms at the speaker assuming pure water [68].

*Speaker characterization:* A data acquisition system (USB-6356, National Instruments, Austin, TX, USA) was used to produce a 0.5 ms signal at a specified frequency; this short duration enables postprocessing analysis to distinguish the direct signal in the measured response from reflections from the tank walls and water surface. This source signal was amplified to drive an underwater speaker (ITC-1042, Gavial, Santa Barbara, CA, USA) positioned 1 m from the reference hydrophone (TC4032, Teledyne Marine, Daytona Beach, FL, USA). The TC4032 has a nominal sensitivity of −164dB re 1V/μPa with a ripple of ±2.45 dB over the band of interest. To ensure the experiment operates in far-field conditions, at least five wavelengths of separation is required, making the measurement valid for frequencies above 7.5 kHz. S1 Appendix - Sect 9 shows additional details of the experimental setup. The data acquisition system is used to generate audio pulses and to measure the responses, enabling automatic calibration and characterization of the device under test. The SPL was measured in response to source signals at frequencies spanning 10–45 kHz. For each frequency, the stimulus amplitude was adjusted to achieve an SPL of 140 dB re 1 μPa. This target level was selected since it can be generated by the output speaker and amplifier with good fidelity in the frequency band of interest while also providing over 30 dB of signal-to-noise ratio for the reference hydrophone and bio-loggers at the receiver location. This process yields a lookup table of signal amplitudes to use for each desired frequency.

**Fig 8:**
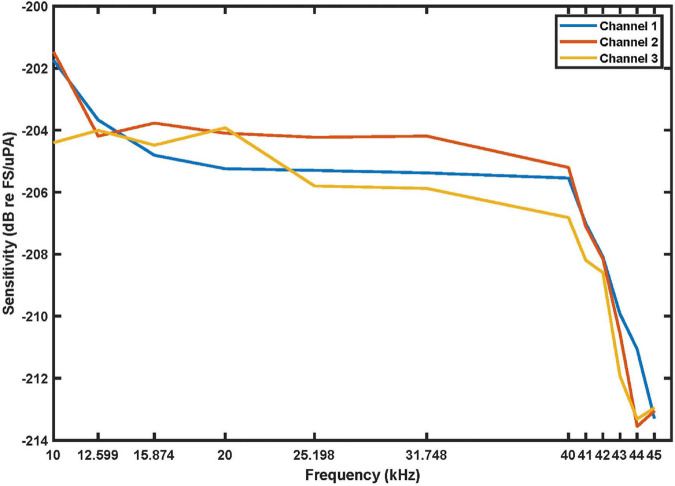
The sensitivity of each hydrophone channel was evaluated at varying frequencies (at 140 dB received SPL).

*Bio-logge hydrophone characterization:* The reference hydrophone is then replaced by a CETI Bio-Logger in the tank at the same location. Recordings are made at each frequency of interest with the stimulus emitted at the target SPL level using the lookup table. From the recorded data, the sensitivity and frequency response of the bio-logger can be determined based on the calibrated reference SPL. Fig 8 shows measured results with a nominal sensitivity of −205dB re FS/μPa. The response rolls off rapidly above 40 kHz due to the ADC’s internal wideband digital low-pass filter. These results are in good agreement with the expected performance of the audio recording subsystem. A characterization of the linearity of the hydrophone with respect to the input sound pressure levels is also shown in S1 Appendix - Sect 10.

### 7.3 Sound event angle of arrival with time difference of arrival

Since a primary motivation for the CETI Bio-Logger is to better understand sperm whale communication, separating vocalizations from distinct whales is valuable for both classical analysis and machine learning pipelines. Towards this end, the CETI Bio-Logger features three strategically placed hydrophones to provide information about angle of arrival and help associate vocalizations with particular whales in the scene.

To validate this capability, a preliminary pipeline was implemented to estimate the angle of arrival of pulses (e.g., sperm whale clicks). The proposed method leverages time difference of arrival (TDoA), which has been explored for underwater localization in a variety of applications [69, 70]. The algorithm first estimates the time difference between when a pulse reaches each hydrophone, then solves for a source direction that is consistent with these latencies given the hydrophone geometry. Underlying this approach is the assumption that audio acquisition from the hydrophones is well-synchronized, has good channel-to-channel isolation, and has good phase/gain matching performance. To validate these characteristics on the CETI Bio-Logger, sinusoidal voltages were applied directly to the hydrophone channels. Applying a signal to only one channel at a time, no crosstalk was detectable except between channel 1 and channel 2 which was at −83dB relative to the signal on the driven channel. Applying a signal to all channels simultaneously, the measured amplitudes were within 0.13 dB of each other and the synchronization was within less than one audio sample. More details are provided in S1 Appendix - Sect 9.6.

To validate the potential for enabling angle of arrival estimation using TDoA, tests were performed in a water tank and in the open ocean. In each case, the bio-logger was held underwater at $0^{\circ }$, $90^{\circ }$, $180^{\circ }$, and $270^{\circ }$ relative to an underwater speaker. In the tank tests, the speaker was 1 m from the bio-logger and played a 10 kHz pulse at each target angle. In open ocean tests, the speaker was 28 m from the bio-logger and played a sequence of five previously recorded sperm whale clicks at each target angle. An initial post-processing pipeline was then implemented as a proof of concept for angle of arrival directionality inference; it consists of isolating each pulse or click, computing the time difference of arrival for each hydrophone, and finally estimating the audio source direction. Note that this estimate is computed within the plane of the bio-logger, as defined by the locations of the three hydrophones.

The average absolute value of the error across all test angles in the water tank and in the open ocean was $6.7^{\circ }\;\pm \;7.6^{\circ }$ and $17.1^{\circ }\;\pm \;20.6^{\circ }$, respectively. To estimate the expected angular resolution of the current bio-logger configuration, each pair of hydrophones can be considered independently and the error in inferred angle when shifting a signal by a single sample and only using these two hydrophones can be considered; taking into account the speed of sound, the sampling rate, and the hydrophone placements, this yields an average expected angular resolution of approximately $10.5^{\circ }$ across all three hydrophone pairs. The results with the preliminary processing pipeline are therefore promising for validating the expected directional capabilities of the current bio-logger configuration. Note that for open ocean tests, the bio-logger was manually rotated and thus some error is also expected in the true angle of each test.

The inclusion of three hydrophones allowed for unambiguous 2D angle of arrival solutions; if only two hydrophones were used, TDoA algorithms would be unable to differentiate between candidate source angles reflected about the line connecting the hydrophones. The current algorithm could be extended to 3D scenarios in the future, in which case there would be an ambiguity between candidate angles above or below the plane of the hydrophones; the assumption of a tagged animal and the bio-logger itself being on one side of this plane may help resolve this ambiguity.

### 7.4 APRS characterization in Dominica

To characterize the capabilities of the recovery system around the island of Dominica where the bio-loggers are currently deployed, the system was configured to poll the GPS module once every minute. If a GPS location was obtained, an APRS message with a comment containing a transmission index was broadcast and the transmission index was incremented. How often the recovery system failed to obtain GPS lock can be determined from the difference in the overall time interval of the test and the transmission indices in the comments received. The probability of a message being received can also be ascertained from the number of transmissions from the recovery system and the number of logged messages on APRS-IS.

As shown in S1 Appendix - Sect 11, the success rate of messages received and relayed by the existing APRS towers on the island varies between 19% and 30%. Although this success rate is low, sporadic messages are sufficient for an operator to estimate the bio-logger’s location and infer that it is still on a whale or initiate a bio-logger retrieval mission. Also, note that this rate of APRS message receipt is independent of the bio-logger’s ability to log GPS locations; every available GPS location can still be saved and used for post-mission analysis.

The message success rate depends on several factors including the distance from the antenna and the topography of the environment. During the characterization experiments on a boat, the farthest successful message was 73 km Northwest from the receiving antenna. For more details and to better visualize APRS coverage, a heat map of the message success rate is shown in S16 Fig. Results are promising for leveraging Dominica’s APRS environment as a reliable recovery method.

Versions of the CETI Bio-Logger deployed in Dominica are currently being located using these APRS messages with GPS information, and by subsequently using a directional antenna to listen for the VHF fish tracker pings upon closer approach to the recovery area.

## 8 Experimental results: Sperm whale deployments

The CETI Bio-Logger has been used to successfully monitor the communication and behavior of wild sperm whales in the open ocean. In particular, families of whales in the Eastern Caribbean off the coast of Dominica have been tagged and observed. These tests validate the capabilities of the bio-logger including high-quality audio recording, behavioral and environmental monitoring, adhesion, and robustness.

Over the course of one year, eight unique instances of the CETI Bio-Logger have been deployed on 10 sperm whales as summarized in Table 1. They have recorded approximately 20 hours of whale activity, including 13 total hours below 50 m. The data includes 19.5 dives averaging 40min±13min and reaching depths of 645m±205m (maximum 967 m).

**Table 1:**
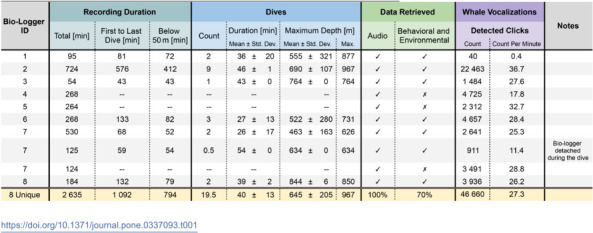
Summary of Successful Bio-Logger Deployments.

**Fig 9:**
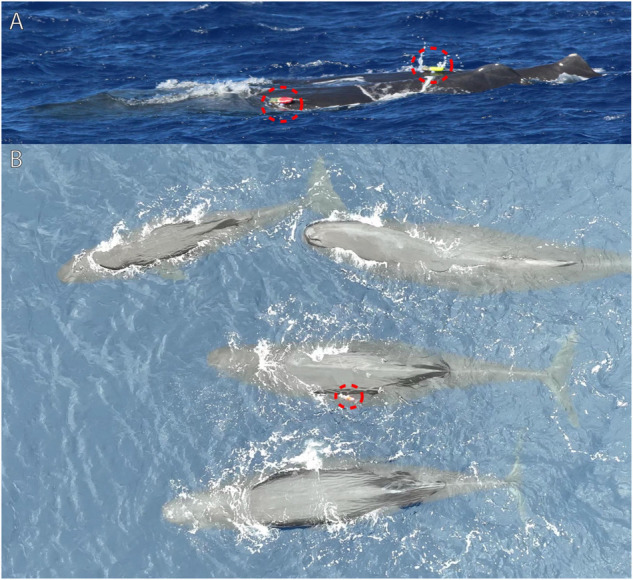
Using on-whale bio-loggers to record acoustic, behavioral, and environmental data during whale interactions provides valuable data for studying their communication. A: Two tagged whales socialize with each other. B: A single tagged whale socializes with other whales.

Examples of successful deployment are shown in Fig 9. These demonstrate scenarios with multiple whales socializing, which are useful for studying their communication and interactions. Fig 9A in particular represents a highly valuable scenario of two whales being tagged while interacting with each other; this ensures high-quality audio and behavioral data from both focal whales, as well as audio from surrounding whales.

Throughout the presented deployments, the bio-loggers experienced a range of challenging conditions that tested robustness in real-world settings. During the seven deployments in Table 1 that successfully recorded behavioral and environmental data, the maximum pressure was approximately 97.5 bar and the ambient temperature ranged from approximately $4.7^{\circ }\;\text{C}$ to $42.4^{\circ }\;\text{C}$. In addition, the bio-loggers experienced maximum linear accelerations and angular velocities of 54.0 m*s*^2^ and 670.2 degs, respectively, during periods inferred to be attached to a whale, and 80.9 m*s*^2^ and 1 466.3 degs, respectively, when including additional time between bio-logger startup and shutdown.

Regarding the deployments listed in Table 1 where behavioral and environmental data were not retrieved, it is suspected that there was water ingress around the depth sensor. This in turn caused an electrical short-circuit within the I2C communication bus, and compromised data from all sensors on that bus. Subsequent iterations of the mechanical design have aimed to address this issue, and this failure mode has not been observed in more recent deployments.

The following sections further discuss the multimodal data recorded by the bio-loggers. The deployment referenced in Table 1 as bio-logger ID 6 and featured in Fig 9B will be used as a running example.

### 8.1 Recorded multimodal data and system performance

**Fig 10:**
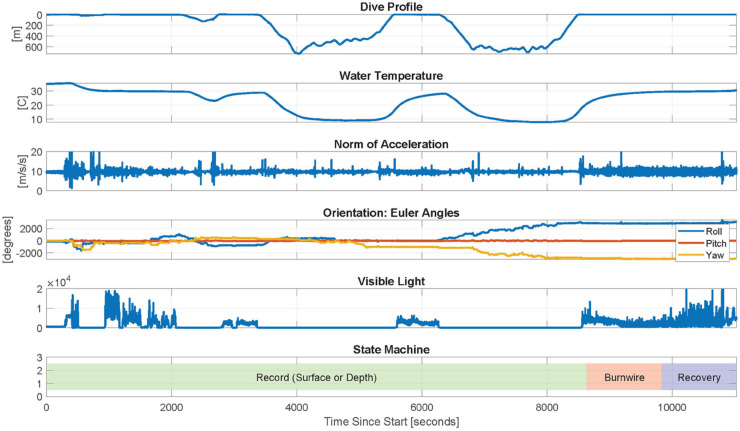
Sensor data is synchronously recorded from environmental and behavioral sensors throughout a deployment.

Throughout a deployment, the bio-logger acquires and logs timestamped data from multiple behavioral and environmental sensors at varying sampling rates. This yields a multimodal dataset from each deployment with synchronized sensor streams, as exemplified in Fig 10. It can be seen that the whale performed a shallow dive shortly after being tagged, followed by two deep dives with a maximum depth of 731 m. The burnwire triggered the bio-logger’s release near the surface approximately 133 min after deployment.

The pressure, temperature, and light sensors provide information about the ambient conditions while the accelerometer, gyroscope, and magnetometer provide information about the whale’s motions. The IMU streams are also processed onboard to estimate the whale’s orientation.

The data also includes information about the bio-logger itself, including battery and system health. During this example deployment, the CPU usage across all cores averaged 35.5%±3.3% with a maximum of 44.6%, and the core dedicated to audio acquisition had steady usage at 22.0%±0.3%. The CPU temperature averaged $39.2^{\circ }\;\text{C}\;\pm \;8.0^{\circ }\;\text{C}$ with a maximum of $53.1^{\circ }\;\text{C}$, which are well below the recommended limit of $85^{\circ }\;\text{C}$. The RAM usage averaged 89.8%±1.7% with a maximum of 94.2%. More information is shown in S1 Appendix - Sect 8. These results indicate that the software successfully leveraged the limited computational resources to maintain robust data acquisition at desired sampling rates. The behavioral and environmental sensors used approximately 41 MB per hour, for a total of 91 MB. The audio used approximately 293 MB per hour, for a total of 650 MB. These confirm that the 256 GB SD card allows for a greater recording capacity than the current battery and adhesion limits. Finally, the state machine also transitioned between initialization, surface recording, depth recording, burnwire, and recovery states as expected to manage resources.

### 8.2 Recorded audio

Audio was successfully recorded throughout all deployments listed in Table 1. The log data indicates that no audio pipeline overflows were detected for bio-logger IDs 3 through 8; this logging capability was not yet added in the first two deployments, but no errors have been detected during post-processing.

**Fig 11:**
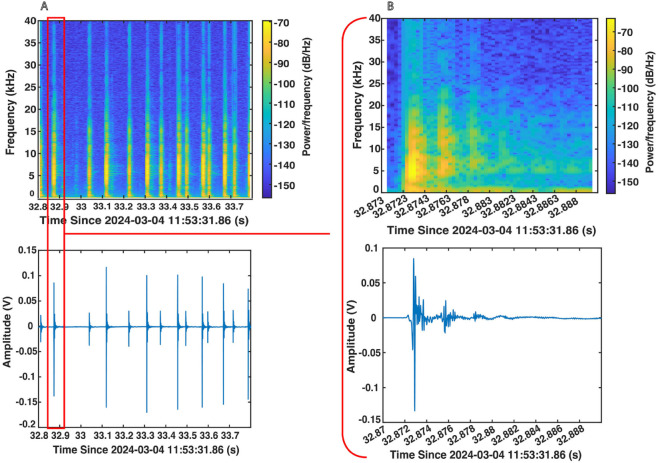
The bio-logger successfully recorded sperm whale vocalizations. A: Multiple echolocation clicks are evident as vertical lines in the spectrogram and waveform. B: The spectrogram and waveform of a single selected click help illustrate the frequency content distribution and the expected multi-pulse structure.

Sample audio segments demonstrating the successful recording of sperm whale clicks are shown in Fig 11. The spectrograms indicate that the expected frequency content was successfully captured, with dominant bands around 5–10 kHz but extending up to approximately 40 kHz. Overlapping groups of clicks, known as codas, are clearly discernible from the focal whale (larger amplitudes) as well as more distant whales (smaller amplitudes) as illustrated in Fig 11A. The bio-logger audio thus supports the analysis of interactions between socializing whales. As seen in Fig 11B, individual clicks also demonstrate the expected multi-pulse structure caused by reflections of the sound in the whale’s head; this can be useful information for inferring the size of the whale and, if sufficient prior field data is available, the whale’s identity.

To explore these features on a larger scale, a click detection algorithm was applied to the recorded audio from all 10 deployments. It is designed to detect echolocation clicks in particular, using a clustering approach based on the expected multi-pulse structure of clicks [71]. Across all deployments, it detected 46 660 clicks averaging 27.3 clicks per minute; results for each deployment are shown in Table 1. Note that communication clicks in particular, and their grouping into codas, can be detected in the future by dedicated pipelines to support communication analysis.

These results validate that the bio-loggers successfully recorded whale vocalizations and interactions, facilitating the curation of large datasets that can be used to extract insights about their communication. Further analysis will be conducted to identify characteristics such as frequency patterns of individual clicks [72] and to group clicks into codas that can be categorized according to their structure for communication analysis [37].

## 9 Considerations for extensibility and scalability

The experimental results demonstrate that the CETI Bio-Logger successfully monitored sperm whales in the open ocean. These operations can be scaled in the future to support larger datasets and more continuous monitoring. A key consideration in this regard is enabling more research groups to fabricate and learn to use these recording devices – to make these technologies more accessible and not require expert design knowledge. Making the hardware and software for the CETI Bio-Logger open-source, including details on the fabrication, aims to take a step towards democratizing such knowledge and encouraging more widespread adoption.

Additional considerations for scaling sperm whale monitoring span deployment, recovery, and data processing pipelines. Intensive manual labor is currently required to find whales, deploy bio-loggers, and locate recoverable bio-loggers. Current practices include steps such as limiting bio-logger adhesion duration to ensure that the whale does not travel too far away from a home base, resulting in an unrecoverable bio-logger. The presented drone-based deployments, the potential for automated recoveries, and scalable data ingestion scripts are promising for addressing such challenges in the future.

An important avenue for scalability also lies in the potential to adapt the bio-logger for monitoring other marine species. The hardware and software have been designed with the aim of enabling future adaptations in this regard. Audio recording can be configured via software, with possible settings spanning up to four synchronized channels, up to 24 bits of resolution, and up to 192 kHz sampling rates; this can thus support a wide range of target acoustic properties. The use of hobbyist-level computational infrastructure featuring a well-documented operating system also facilitates changes to recording behavior based on real-time sensor readings, or adding additional sensors (especially ones with common communication protocols such as I2C or SPI).

Finally, although it may require greater design knowledge, the mechanical structure can be adapted to support different attachments or target specifications. These could include using the same electronics within a body that has a collar-based attachment mechanism instead of suction cups, which may be more amenable to animals with fur or rough skins. In addition, adjustments could be made to reduce the bio-logger size and weight for smaller animals; in particular, given that sperm whales are among the deepest-diving mammals, the bio-logger can be simplified for animals that do not dive to such extreme depths. A lighter and smaller flotation structure can be used instead of syntactic foam, and a lighter dry or oil-filled housing could be used instead of epoxy potting. The size and weight of the battery, which is tightly coupled with the size of the flotation material, could also be adjusted based on target recording durations and the expected amount of wireless communication.

## 10 Conclusion

In this study, we describe a new open-source bio-logger which can be used to study animal vocalizations and behaviors. Its current implementation is designed for whale communication studies, and its size and weight precludes its use on small species, but its component parts and systems could be generalized for studies on other species.

The bio-logger includes four audio channels (three of which are currently in use) capable of recording simultaneously at high sampling rates (96 kHz or above); this aims to help reduce sound source ambiguities when several whales are vocalizing, compared to bio-loggers equipped with fewer hydrophones. Preliminary laboratory and in-field experiments demonstrate the potential for audio source angle of arrival estimation.

This audio data is accompanied by an extensible suite of behavioral and environmental sensors which provide important contextual information. These currently include depth, motion, temperature, and light.

The CETI Bio-Logger also includes multiple methods of localization and recovery, including a VHF beacon and GPS. GPS data can be transmitted via APRS, which leverages existing world-wide mesh networks from the amateur radio community. This aims to provide an inexpensive and useful way for researchers to deploy and recover bio-loggers within tens of kilometers of shore.

A critical goal in the development of the CETI Bio-Logger is making it open-source to promote adoption within the marine mammal research community and beyond. For example, the bio-logger’s main electronics board can be reused for other behavioral or vocalization studies ranging from marine to terrestrial environments.

Future research on the CETI Bio-Logger will focus on making the bio-logger smaller, reducing its energy consumption, integrating additional features such as video, enhancing adhesion for longer-duration deployments, and developing new deployment or recovery techniques.

Together, these capabilities and community benefits aim to encourage more widespread use of sensor bio-loggers for studying marine or terrestrial animals. Such a movement can help bolster efforts to curate large multimodal datasets for machine learning, and fuel data-driven approaches to creating more symbiotic and resilient relationships with our surrounding ecosystems.

## Supporting information

S1 Appendix Supporting information. (PDF)

## References

[1] Bakker K. The sounds of life: how digital technology is bringing us closer to the worlds of animals and plants. Princeton University Press; 2022.

[2] Andreas J, Beguš G, Bronstein MM, Diamant R, Delaney D, Gero S, et al. Toward understanding the communication in sperm whales. iScience. 2022;25(6):104393. doi: 10.1016/j.isci.2022.104393 35663036 PMC9160774

[3] Noda T, Koizumi T, Yukitake N, Yamamoto D, Nakaizumi T, Tanaka K, et al. Animal-borne soundscape logger as a system for edge classification of sound sources and data transmission for monitoring near-real-time underwater soundscape. Sci Rep. 2024;14(1):6394. doi: 10.1038/s41598-024-56439-x 38493174 PMC10944488

[4] Chung H, Lee J, Lee WY. A review: marine bio-logging of animal behaviour and ocean environments. Ocean Sci J. 2021;56(2):117–31. doi: 10.1007/s12601-021-00015-1

[5] Duarte CM, Chapuis L, Collin SP, Costa DP, Devassy RP, Eguiluz VM, et al. The soundscape of the Anthropocene ocean. Science. 2021;371(6529):eaba4658. doi: 10.1126/science.aba4658 33542110

[6] Payne NL, Taylor MD, Watanabe YY, Semmens JM. From physiology to physics: are we recognizing the flexibility of biologging tools?. J Exp Biol. 2014;217(Pt 3):317–22. doi: 10.1242/jeb.093922 24477606

[7] McIntyre T. Trends in tagging of marine mammals: a review of marine mammal biologging studies. African Journal of Marine Science. 2014;36(4):409–22. doi: 10.2989/1814232x.2014.976655

[8] Harcourt R, Sequeira AMM, Zhang X, Roquet F, Komatsu K, Heupel M, et al. Animal-borne telemetry: an integral component of the ocean observing toolkit. Front Mar Sci. 2019;6. doi: 10.3389/fmars.2019.00326

[9] Holton MD, Wilson RP, Teilmann J, Siebert U. Animal tag technology keeps coming of age: an engineering perspective. Philos Trans R Soc Lond B Biol Sci. 2021;376(1831):20200229. doi: 10.1098/rstb.2020.0229 34176328 PMC8237169

[10] March D, Boehme L, Tintoré J, Vélez-Belchi PJ, Godley BJ. Towards the integration of animal-borne instruments into global ocean observing systems. Glob Chang Biol. 2020;26(2):586–96. doi: 10.1111/gcb.14902 31675456 PMC7027834

[11] Williams CL, Ponganis PJ. Diving physiology of marine mammals and birds: the development of biologging techniques. Philos Trans R Soc Lond B Biol Sci. 2021;376(1830):20200211. doi: 10.1098/rstb.2020.0211 34121464 PMC8200650

[12] CETI Bio-Logger website. [cited 2025 Nov 1]. https://www.projectceti.org/research/

[13] CETI. Project CETI. 2024. https://www.projectceti.org/

[14] Amano M, Yoshioka M. Sperm whale diving behavior monitored using a suction-cup-attached TDR tag. Mar Ecol Prog Ser. 2003;258:291–5. doi: 10.3354/meps258291

[15] White C, Miller JH, Potty GR, Johnson M. Dynamic tracking of free-swimming whale groups using digital acoustic recording tags. The Journal of the Acoustical Society of America. 2008;123(5_Supplement):3362–3362. doi: 10.1121/1.2933957

[16] Johnson MP, Tyack PL. A digital acoustic recording tag for measuring the response of wild marine mammals to sound. IEEE J Oceanic Eng. 2003;28(1):3–12. doi: 10.1109/joe.2002.808212

[17] CATS tag. https://cats.is/. Accessed 2024 March 18.

[18] Burgess WC, McGuire TL. A novel approach to measurement of underwater sound levels in a dangerous tidal fjord using a miniature self-contained acoustic recorder. Journal of the Acoustical Society of America. 2016;140(4_Supplement):3182–3182. doi: 10.1121/1.4970005

[19] Kleivane L, Kvadsheim PH, Bocconcelli A, Øien N, Miller PJO. Equipment to tag, track and collect biopsies from whales and dolphins: the ARTS, DFHorten and LKDart systems. Anim Biotelemetry. 2022;10(1). doi: 10.1186/s40317-022-00303-0

[20] Irvine LM, Winsor MH, Follett TM, Mate BR, Palacios DM. An at-sea assessment of Argos location accuracy for three species of large whales, and the effect of deep-diving behavior on location error. Anim Biotelemetry. 2020;8(1). doi: 10.1186/s40317-020-00207-x

[21] Wiley DN, Zadra CJ, Friedlaender AS, Parks SE, Pensarosa A, Rogan A, et al. Deployment of biologging tags on free swimming large whales using uncrewed aerial systems. R Soc Open Sci. 2023;10(4):221376. doi: 10.1098/rsos.221376 37090967 PMC10113809

[22] Whitford M, Klimley AP. An overview of behavioral, physiological, and environmental sensors used in animal biotelemetry and biologging studies. Anim Biotelemetry. 2019;7(1). doi: 10.1186/s40317-019-0189-z

[23] Rudd JL, Bartolomeu T, Dolton HR, Exeter OM, Kerry C, Hawkes LA, et al. Basking shark sub-surface behaviour revealed by animal-towed cameras. PLoS One. 2021;16(7):e0253388. doi: 10.1371/journal.pone.0253388 34320007 PMC8318306

[24] Alex Shorter K, Shao Y, Ojeda L, Barton K, Rocho-Levine J, van der Hoop J, et al. A day in the life of a dolphin: using bio-logging tags for improved animal health and well-being. Marine Mammal Science. 2017;33(3):785–802. doi: 10.1111/mms.12408

[25] Houstin A, Zitterbart DP, Winterl A, Richter S, Planas-Bielsa V, Chevallier D, et al. Biologging of emperor penguins-attachment techniques and associated deployment performance. PLoS One. 2022;17(8):e0265849. doi: 10.1371/journal.pone.0265849 35925903 PMC9352057

[26] Linsky JMJ, Wilson N, Cade DE, Goldbogen JA, Johnston DW, Friedlaender AS. The scale of the whale: using video-tag data to evaluate sea-surface ice concentration from the perspective of individual Antarctic minke whales. Anim Biotelemetry. 2020;8(1). doi: 10.1186/s40317-020-00218-8

[27] Fontes J, Macena B, Solleliet-Ferreira S, Buyle F, Magalhães R, Bartolomeu T, et al. Correction: The advantages and challenges of non-invasive towed PILOT tags for free-ranging deep-diving megafauna. Anim Biotelemetry. 2023;11(1). doi: 10.1186/s40317-023-00321-6

[28] Urbano F, Cagnacci F, Calenge C, Dettki H, Cameron A, Neteler M. Wildlife tracking data management: a new vision. Philos Trans R Soc Lond B Biol Sci. 2010;365(1550):2177–85. doi: 10.1098/rstb.2010.0081 20566495 PMC2894960

[29] Tønnesen P, Gero S, Ladegaard M, Johnson M, Madsen PT. First-year sperm whale calves echolocate and perform long, deep dives. Behav Ecol Sociobiol. 2018;72(10). doi: 10.1007/s00265-018-2570-y

[30] Saddler MR, Bocconcelli A, Hickmott LS, Chiang G, Landea-Briones R, Bahamonde PA, et al. Characterizing Chilean blue whale vocalizations with DTAGs: a test of using tag accelerometers for caller identification. J Exp Biol. 2017;220(Pt 22):4119–29. doi: 10.1242/jeb.151498 28883086

[31] McDonald BI, Johnson M, Madsen PT. Dive heart rate in harbour porpoises is influenced by exercise and expectations. J Exp Biol. 2018;221(Pt 1):jeb168740. doi: 10.1242/jeb.168740 29122951

[32] Warner GA, Dosso SE, Hannay DE. Bowhead whale localization using time-difference-of-arrival data from asynchronous recorders. J Acoust Soc Am. 2017;141(3):1921. doi: 10.1121/1.4978438 28372102

[33] Alexandri T, Diamant R. Detection and characterization of ship underwater radiated narrowband noise. Computer Networks. 2024;248:110480. doi: 10.1016/j.comnet.2024.110480

[34] Sonne C, Jepson PD, Desforges J-P, Alstrup AKO, Olsen MT, Eulaers I, et al. Pollution threatens toothed whales. Science. 2018;361(6408):1208. doi: 10.1126/science.aav2403 30237348

[35] Tulloch VJD, Plagányi ÉE, Brown C, Richardson AJ, Matear R. Future recovery of baleen whales is imperiled by climate change. Glob Chang Biol. 2019;25(4):1263–81. doi: 10.1111/gcb.14573 30807685 PMC6850638

[36] Leitao A, Lucas M, Poetto S, Hersh TA, Gero S, Gruber DF. Evidence of social learning across symbolic cultural barriers in sperm whales. eLife. 2024;13.

[37] Sharma P, Gero S, Payne R, Gruber DF, Rus D, Torralba A, et al. Contextual and combinatorial structure in sperm whale vocalisations. Nat Commun. 2024;15(1):3617. doi: 10.1038/s41467-024-47221-8 38714699 PMC11076547

[38] Goldwasser S, Gruber D, Kalai AT, Paradise O. A theory of unsupervised translation motivated by understanding animal communication. Advances in Neural Information Processing Systems. 2024;36.

[39] Beguš G, Leban A, Gero S. Approaching an unknown communication system by latent space exploration and causal inference. arXiv preprint 2023. https://arxiv.org/abs/2303.10931

[40] Youngblood M. Language-like efficiency in whale communication. PsyArXiv preprint 2024. 10.1126/sciadv.ads6014 PMC11797547 39908378

[41] Madsen PT, Payne R, Kristiansen NU, Wahlberg M, Kerr I, Møhl B. Sperm whale sound production studied with ultrasound time/depth-recording tags. J Exp Biol. 2002;205(Pt 13):1899–906. doi: 10.1242/jeb.205.13.1899 12077166

[42] Goold JC, Jones SE. Time and frequency domain characteristics of sperm whale clicks. J Acoust Soc Am. 1995;98(3):1279–91. doi: 10.1121/1.413465 7560502

[43] Møhl B, Wahlberg M, Madsen PT, Heerfordt A, Lund A. The monopulsed nature of sperm whale clicks. J Acoust Soc Am. 2003;114(2):1143–54. doi: 10.1121/1.1586258 12942991

[44] Watwood SL, Miller PJO, Johnson M, Madsen PT, Tyack PL. Deep-diving foraging behaviour of sperm whales (Physeter macrocephalus). J Anim Ecol. 2006;75(3):814–25. doi: 10.1111/j.1365-2656.2006.01101.x 16689963

[45] Miller PJO, Aoki K, Rendell LE, Amano M. Stereotypical resting behavior of the sperm whale. Curr Biol. 2008;18(1):R21-3. doi: 10.1016/j.cub.2007.11.003 18177706

[46] Teloni V, Mark JP, Patrick MJO, Peter MT. Shallow food for deep divers: dynamic foraging behavior of male sperm whales in a high latitude habitat. Journal of Experimental Marine Biology and Ecology. 2008;354(1):119–31. doi: 10.1016/j.jembe.2007.10.010

[47] Systems ES. Guide to syntactic machining. [cited 2024 June 22]. https://ess.globecomposite.com/technical-guides/

[48] Systems ES. Syntactic foam case studies. [cited 2024 June 22]. https://ess.globecomposite.com/case-studies/

[49] Andrews RD, Baird RW, Calambokidis J, Goertz CEC, Gulland FMD, Heide-Jorgensen MP, et al. Best practice guidelines for cetacean tagging. J Cetacean Res Manage. 2019;20(1):27–66. doi: 10.47536/jcrm.v20i1.237

[50] Bell MA, Becker KP, Wood RJ. Injection molding of soft robots. Advanced Materials Technologies. 2022;7(1):2100605.

[51] Hernandez AM, Sandoval JA, Yuen MC, Wood RJ. Stickiness in shear: stiffness, shape, and sealing in bioinspired suction cups affect shear performance on diverse surfaces. Bioinspir Biomim. 2024;19(3):10.1088/1748-3190/ad2c21. doi: 10.1088/1748-3190/ad2c21 38528733

[52] KELLER Druckmesstechnik AG. Data sheet: piezoresistive OEM pressure transmitters with I2C interface and embedded signal conditioning, series 4LD...9LD. 2021.

[53] Saunders PM. Practical conversion of pressure to depth. J Phys Oceanogr. 1981;11(4):573–4. doi: 10.1175/1520-0485(1981)011<0573:pcoptd>2.0.co;2

[54] CEVA, Inc. 1000-3927 - BNO08X datasheet. 2023.

[55] Dahl PH, Miller JH, Cato DH, Andrew K. Underwater ambient noise. Acoustics Today. 2007;3(1):23–33.

[56] Wenz GM. Acoustic ambient noise in the ocean: spectra and sources. J Acoust Soc Am. 1962:1936–56.

[57] Kester W. Analog devices MT-022 ADC architectures III: sigma-delta ADC basics. 2008.

[58] Analog Devices, Inc. Data sheet: 8-/4- channel, 24-bit, simultaneous sampling ADC. Analog Devices, Inc. 2018.

[59] Vogt DM, Pagani S, Gonzalez-Peltier Z, Gero S, Gruber DF, Wood RJ. Drone-based application of whale tags: a “tap-and-go” approach for scientific animal-borne investigations. PLoS One. 2025;20(8):e0328037. doi: 10.1371/journal.pone.0328037 40802581 PMC12348971

[60] Warren V, Miller P, Tyack P. Short-term responses of sperm whales Physeter macrocephalus to the attachment of suction cup tags. Mar Ecol Prog Ser. 2020;645:219–34. doi: 10.3354/meps13344

[61] Rodriguez-Garavito C, Gallant J. Listening to the more-than-human world: Legal & ethical principles for nonhuman animal communication technologies. 2024. [cited 2024 Dec 18]. https://www.openglobalrights.org/listening-to-the-more-than-human-world-legal-and-ethical-principles-for-nonhuman-animal-communication-technologies/

[62] Rodriguez-Garavito C, Gruber D. What if we understood what animals are saying? The legal impact of AI-assisted studies of animal communication. Ecology Law Quarterly. 2024.

[63] APRS Working Group. APRS Protocol Reference: Protocol Version 1.0. 2000.

[64] Bruninga B. APRS-IS. [cited 2024 June 14]. https://aprs-is.net/

[65] APRS. [cited 2024 June 14]. https://aprs.fi/

[66] Hernandez AM, Sandoval JA, Yuen MC, Wood RJ. Bioinspired surface structures for added shear stabilization in suction discs. Scientific Reports. 2024. 10.1038/s41598-024-82221-0 PMC11704247 39762238

[67] Levin PA. Calibration of hydrophones. Bruel and Kjaer Technical Review. 1973.

[68] Bilaniuk N, Wong GSK. Speed of sound in pure water as a function of temperature. The Journal of the Acoustical Society of America. 1993;93(4_Supplement):2306–2306. doi: 10.1121/1.406451

[69] Alexandri T, Walter M, Diamant R. A time difference of arrival based target motion analysis for localization of underwater vehicles. IEEE Trans Veh Technol. 2022;71(1):326–38. doi: 10.1109/tvt.2021.3120201

[70] Su X, Ullah I, Liu X, Choi D. A review of underwater localization techniques, algorithms, and challenges. Journal of Sensors. 2020;2020:1–24. doi: 10.1155/2020/6403161

[71] Gubnitky G, Diamant R. Detecting the presence of sperm whales’ echolocation clicks in noisy environments. IEEE/ACM Transactions on Audio, Speech, and Language Processing. 2024.

[72] Beguš G, Sprouse RL, Leban A, Gero S. Vowels and Diphthongs in Sperm Whales. OSF Preprints. 2023.

