# Supplementary material for: An open-source bio-logger for studying cetacean behavior and communication
Source: PLoS One. 2025 Dec 11;20(12):e0337093. doi: 10.1371/journal.pone.0337093 (PMC12697950; doi:10.1371/journal.pone.0337093)
Supplement: S1 Appendix — (PDF) [file pone.0337093.s001.pdf]

S1 Appendix: Supplementary Information

Contents

1 Bio-Logger Specifications **2**

2 Fabrication **4**
2.1 Bio-Logger Body Assembly and Resin Injection 4
2.2 Hydrophones 7
2.3 Suction Cups 8
2.4 Antennae 9

3 Suction Cup Evaluation **10**

4 Bio-Logger Release System **11**

5 Bio-Logger Buoyancy **12**

6 Bio-Logger Pressure Cycles **13**

7 Bio-Logger Power Budget **14**

8 System Resource Consumption **15**

9 Audio System Characterization **16**
9.1 Introduction 16
9.2 Underwater Characterization 16
9.3 Sensitivity and Frequency Response 16
9.4 Electronic Self-Noise and Interference 18
9.5 Equipment List 18
9.6 Bio-Logger Hydrophone Array: Crosstalk, Phase, and Amplitude Matching 19

10 Hydrophone Response to Sound Pressure Level **21**

11 APRS Characterization in Dominica **22**

12 Computational Fluid Dynamics (CFD) **24**

13 Bill of Materials **26**
13.1 General Components 26
13.2 Printed Circuit Boards and Other Electronic Components 28

14 Comparison to Existing Bio-Loggers **30**

1 Bio-Logger Specifications

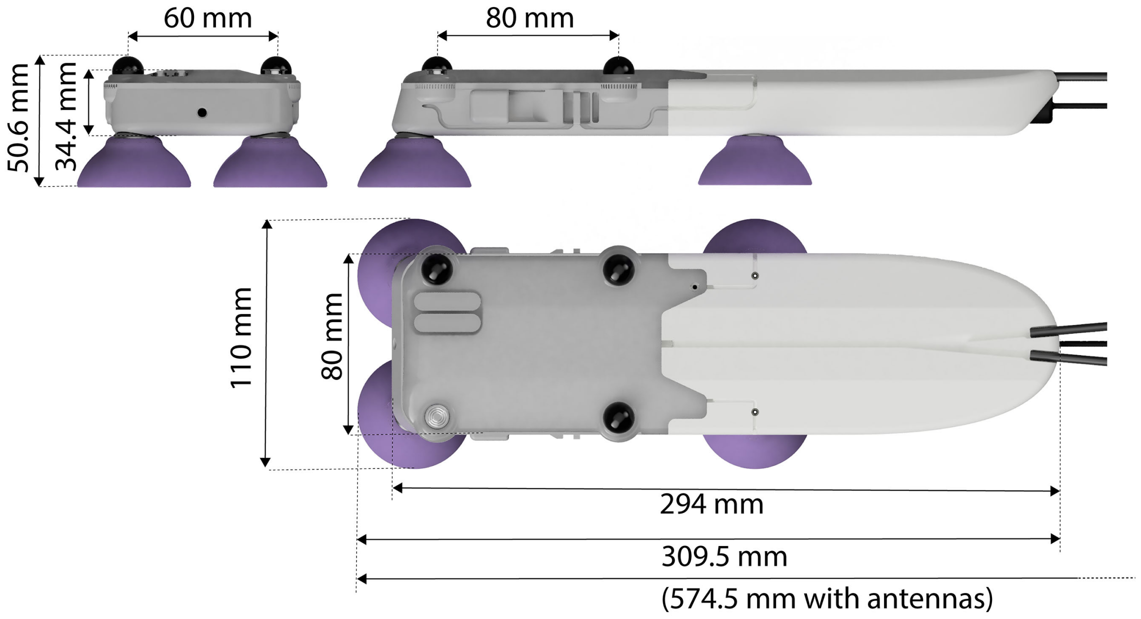

Figure S1: Overall dimensions of the `CETI Bio-Logger`.

**Physical Characteristics and Ratings**	
Dimensions	110 x 50.6 x 574.5 mm (4.33 x 1.99 x 29.7 in)
Weight	0.7 kg (1.54 lbs)
Adhesion to Animal	4 custom suction cups
Maximum Depth	Tested up to 1,000 m
Battery Capacity	2 parallel Li-Ion 5,000 mAh cells (37 Wh)
Price of Parts	$984.77
**Audio Recording**	
Channels	4 implemented in electronics<br>3 hydrophones currently populated
Sampling Rate	96 kHz (up to 192 kHz) per channel
Resolution	16 bits per sample
Sensitivity	-205 dB re FS / 1 µPa
Dynamic Range	96 dB
Storage	Onboard lossless compression
**Sensor Suite**	
Behavior	Depth, motion, and orientation
Environment	Water temperature, visible and IR light<br>GPS locations when available
**Data Formats and Rates**	
Audio	
Uncompressed Data	659.2 MB/hour per channel
Losslessly Compressed Data in `FLAC` Format	≈100 MB/hour per channel
Behavioral and Environmental Sensors	
Combined Data Rate	41.0 MB/hour
Data Format	Human-readable CSV files
**Onboard Computation and Storage**	
Single-Board Computer	Raspberry Pi Zero 2 W<br>1 GHz Quad Core, 512 MB of RAM<br>256 GB microSD card
Signal Routing and Pre-Processing	FPGA: Xilinx XC3S200A-5VQG100C
**Operational Considerations**	
Recording Duration	
Battery Life	Up to 16.8 hours recording
Data Storage	Up to approximately 730 hours
Suction Cups	Programmable release via burnwires
Data Offloading and Programming	
Wireless Interfaces	Wi-Fi 802.11ac, Bluetooth
Wired Interfaces	Ethernet via USB
Deployment and Recovery	
GPS-Based Recovery	GPS locations sent via APRS
VHF-Based Recovery	VHF beacon (standalone fish tracker)
Deployment Methods	Drone-based or pole-based methods

Figure S2: High-level specifications of the `CETI Bio-Logger`. The formatting is based on [1, 2].

2 Fabrication

2.1 Bio-Logger Body Assembly and Resin Injection

An exploded view of the components in the bio-logger is shown in Fig S3. To mold this epoxy and house all bio-logger components, top and bottom halves of the shell are 3D-printed in VeroClear on a PolyJet printer (Object Scholar, Stratasys, Eden Prairie, MN, USA). To manufacture the bio-logger, the bottom shell is first populated with batteries and with electronics and pneumatic tubing that constitute the burnwire system for programmatically releasing bio-logger suction. The top shell is populated with the hydrophones, depth sensor, external connector, and PCBs. The top and bottom shells are then soldered together to connect the batteries and burnwire components to the main PCB. The shells are sealed using epoxy fairing compound (TotalFair, TotalBoat, Bristol, RI, USA). After curing completes, the syntactic foam is inserted into the shell assembly and sealed with epoxy fairing compound. Finally, the bio-logger is potted by injecting translucent epoxy (832C, MG Chemicals, Ontario, Canada) via luer lock inserts at the top and bottom of the sealed bio-logger. The injection process is shown in Fig S4, where the bio-logger is filled with resin from the bottom to the top. The feeding inlet nozzle is driven by a dual-cartridge epoxy gun connected to a static mixing nozzle.

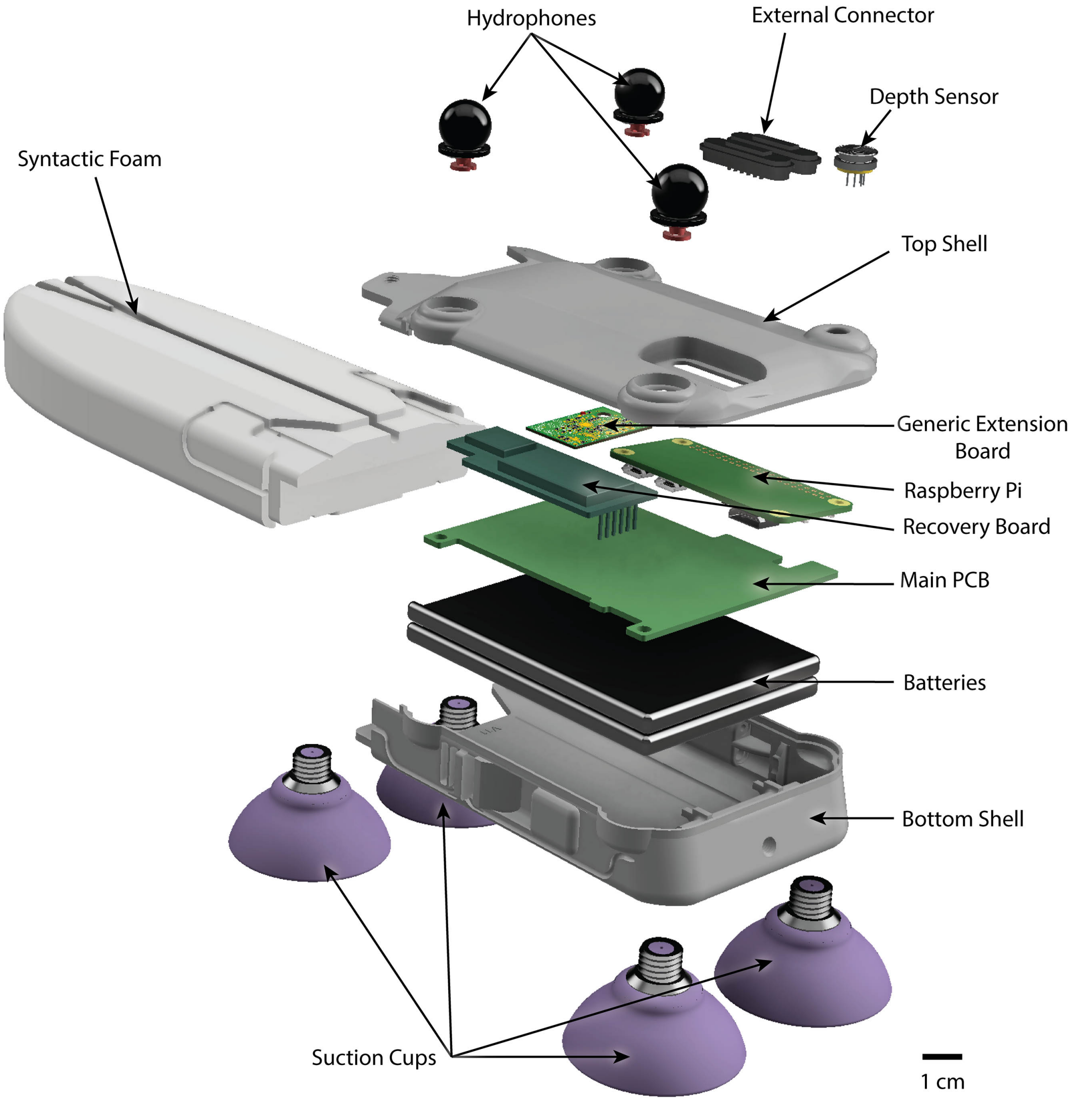

Figure S3: Exploded view of the `CETI Bio-Logger` main components.

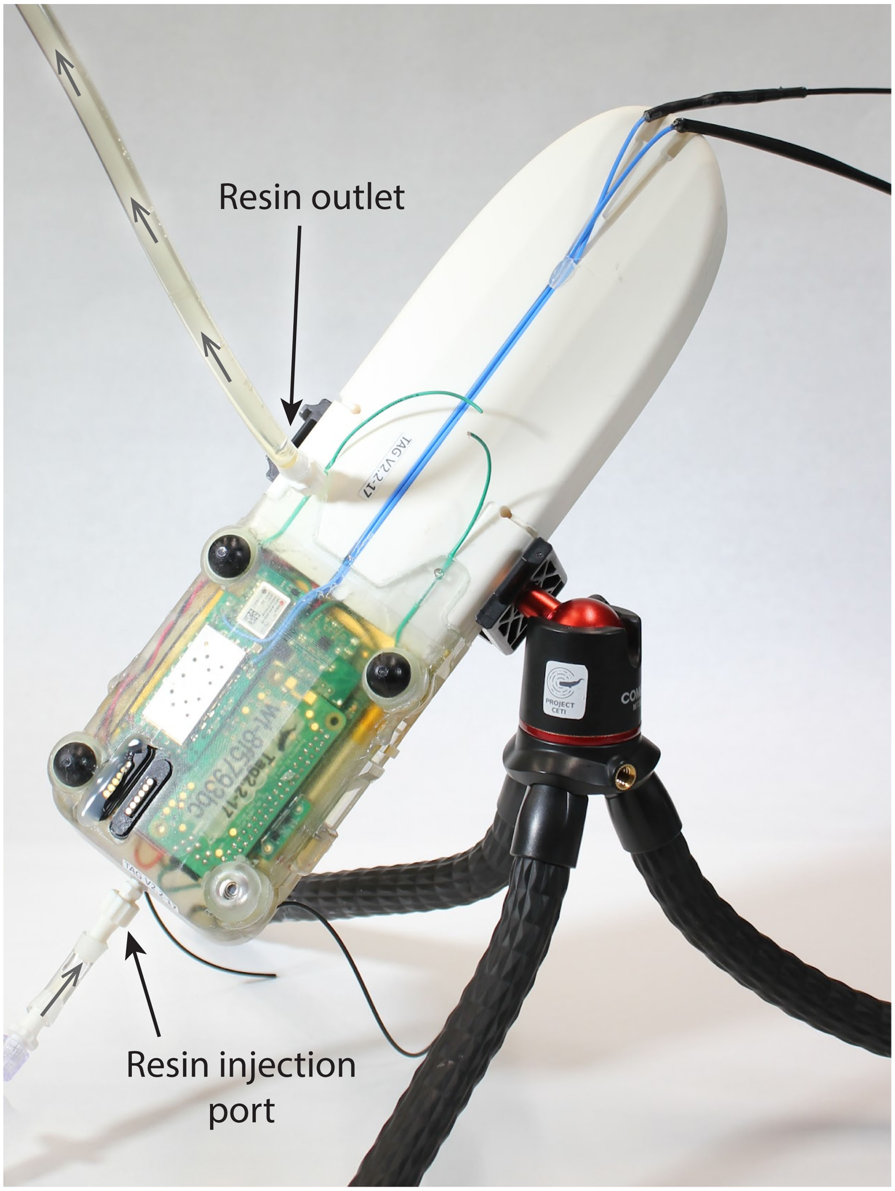

Figure S4: Tag injection with epoxy resin.

2.2 Hydrophones

Fig S5 summarizes the manufacturing process. A coaxial shielded cable is soldered on one end to the piezoelectric element (Fig S5A) (PZT5A, Piezo Hanna, Xianning, Hubei Province, China). A 3D-printed standoff is glued to the base of the sphere to protect the wire and interface with the rest of the bio-logger (Fig S5B).

Hydrophone molds were 3D-printed on a PolyJet in VeroClear and then heated at 60° C for 12 hours to prevent contamination during the curing process [3]. The molds were sprayed with a silicon release agent (Ease Release 200, Mann Release Technologies, Macungie, PA, USA) for easy removal after curing (Fig S5C).

Molds were assembled with the piezoelectric sphere inside and a luer lock interface for injection (Fig S5D). Acoustically transparent rubber (PR-1547 potting compound, PPG Industries, Pittsburgh, PA, USA) part A was heated at 100° C for 30 minutes to decrystalize. Parts A and B were combined at a ratio of 32:100 by weight using a planetary centrifugal mixer (ARE-310, Thinky Mixer, Laguna Hills, CA, USA) and transferred to a 50 mL luer lock syringe. Each hydrophone mold was injected until rubber flowed out of the top (Fig S5E). Caps were secured to the inlets and the molds were placed in a vacuum chamber at -10 psi for one minute to remove air bubbles. More rubber was injected until all bubbles were removed. The hydrophones were cured in a pressure oven at 550 kPa and 80° C for 8 hours.

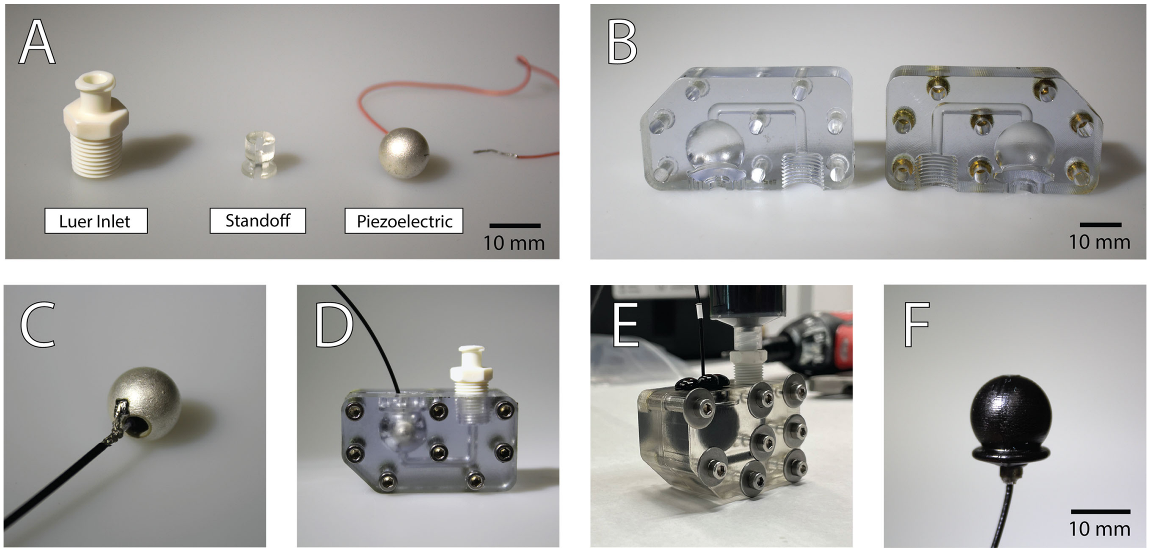

Figure S5: Hydrophone Manufacturing. A: Hydrophone manufacturing components. B: Hydrophone molds. C: Coaxial wire soldered to piezoelectric. D: Hydrophone mold assembled. E: Injection of acoustically transparent rubber. F: Finished `CETI Bio-Logger` hydrophone.

2.3 Suction Cups

Fig S6 shows the manufacturing process for the suction cups. The bottom mold is 3D printed in VeroWhite on a PolyJet to create a smooth surface finish on the inner face of the suction cup. This is essential for suction cup performance. The top mold is made out of Polylactic Acid(PLA) by using a Fused Deposition Modeling (FDM) 3D printer (MK3, Prusa, Czech Republic). The embedded piece is 3D printed in nylon on a Selective Laser Sintering (SLS) printer (Fuse, FormLabs, Somerville MA, USA). The top and bottom molds were sprayed with a silicon release agent (Ease Release 200, Mann Release Technologies, Macungie, PA, USA). The embedded component is threaded into the top mold and the two-piece mold is sealed with screws (Fig S6B). A stainless steel 21-gauge syringe needle (75165A681, McMaster-Carr, Elmhurst, IL, USA) is inserted into the mold through a hole in the top and secured with hot glue (Fig S6C). The molds were injected with a two-part silicon rubber (Smooth-Sil 945, Smooth-On, Macungie, PA, USA) using a static mixing nozzle (Optimixer 33, Nordson, Westlake, OH, USA) until excess flowed out the exit holes (Fig S6D). The suction cups were cured in a pressure oven at 550 kPa and 50° C for two hours.

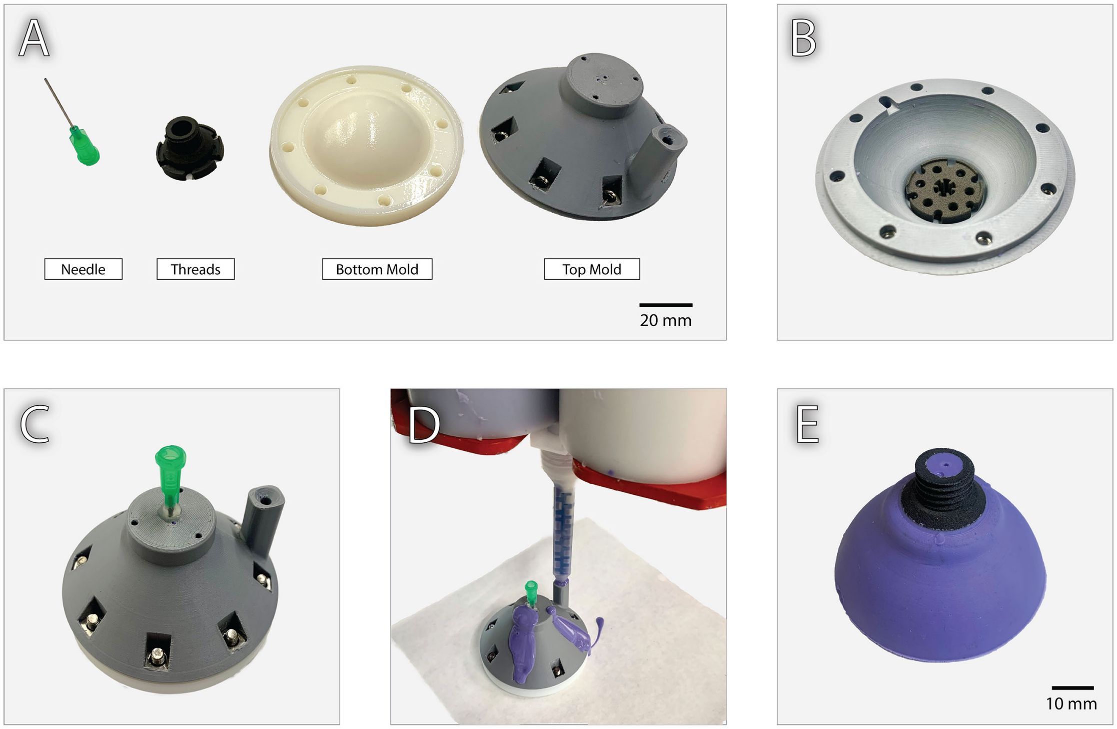

Figure S6: Suction cup manufacturing. A: Injection mold materials. B: Embedded component in top mold. C: Assembled mold with syringe needle. D: Injection of silicon rubber. E: Finished `CETI Bio-Logger` suction cup.

2.4 Antennae

The `CETI Bio-Logger`'s recovery board features two dipole antennae that are mounted at the back of the bio-logger such that they protrude out of the water when the bio-logger is floating. Both antennae are simple dipoles constructed from steel cable. The antenna for receiving GPS is designed to be a 1/4 wavelength (4.6725 cm) at 1.57542 GHz. The antenna for receiving VHF is designed to be a 1/8 wavelength (25.97 cm) at the common APRS frequency in Dominica of 150.05 MHz.

28 AWG coax cable runs from the recovery board potted inside the bio-logger, through a channel in the syntactic foam, and then to these antennae. Solder seal connectors are used to electrically connect the coax core to the dipole antenna. The entire antenna is encased in 1/16 in marine-grade heatshrink, and UV-curable epoxy is used to seal the seams at the ends of the heatshrink.

3 Suction Cup Evaluation

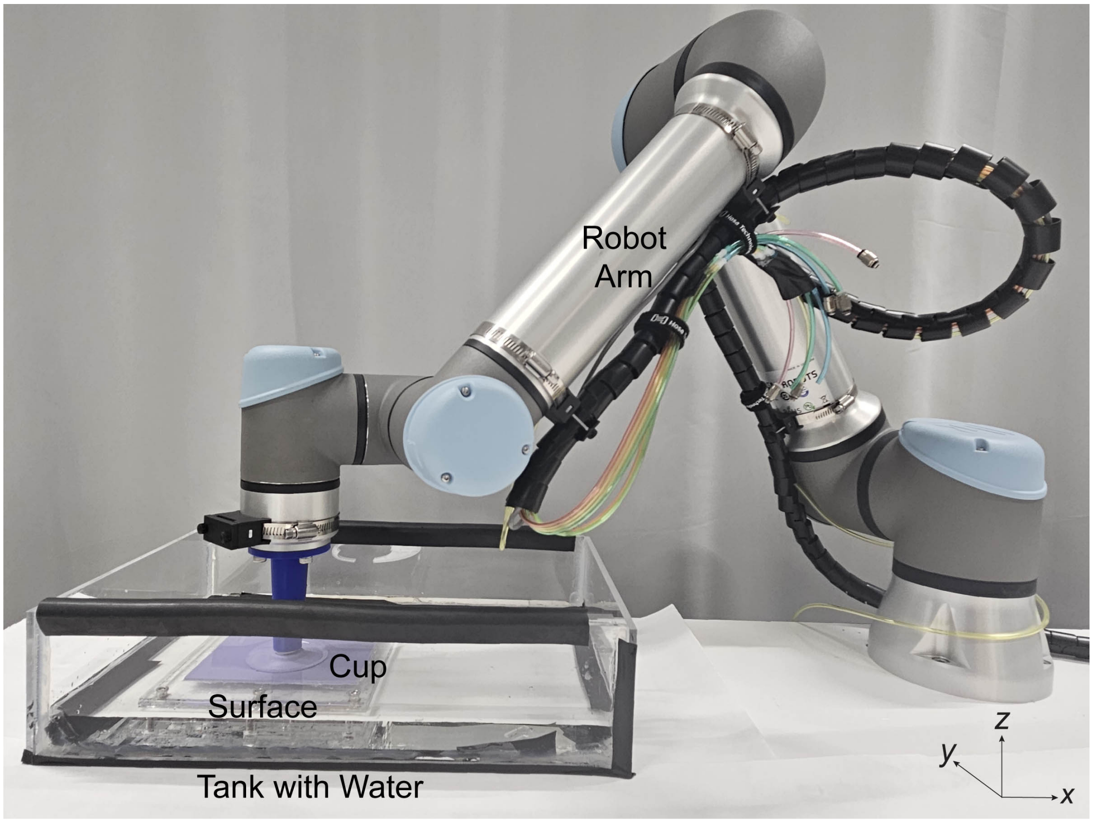

Figure S7: Robot arm setup. When initiated, the robot arm moved the end-effector and cup down towards the test surface (along the z-axis). When contacting the surface, a normal force was applied to the cup until the force sensor reached the designated preload. The arm then reduced the force of the preload to 2 N by moving the end effector away from the surface, thereby creating suction within the chamber. The arm then applied a shear load (along the x-axis) at a constant speed so that the cup was dragged along the surface for a set distance (10 mm). Force (N) and time (s) were recorded. This caption was adapted from [4], where additional information can be found about the arm setup and parameters.

4 Bio-Logger Release System

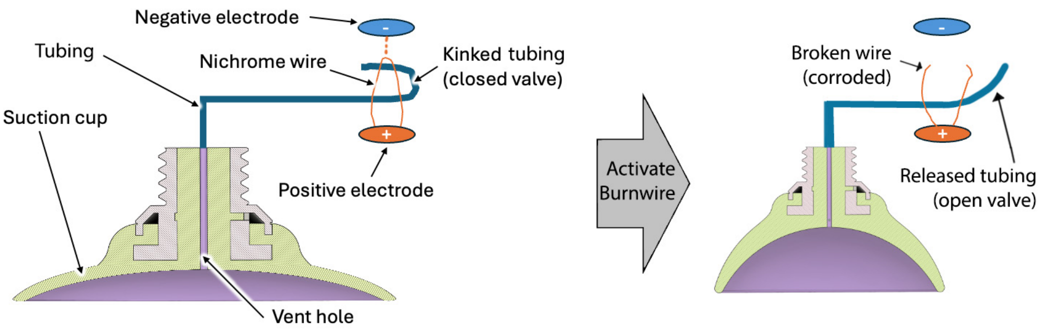

Figure S8: The burnwire mechanism allows the bio-logger to programmatically detach from the whale, triggered by a timer, battery level, or geofencing. The suction cup contains a vent hole connected to a tube, which is kinked at its end and held in place by a Nichrome wire. When voltage is applied to the electrodes, the Nichrome wire undergoes an oxidation reaction and dissolves in the salt water. Once the Nichrome wire breaks, the tube straightens, releasing the suction cup.

5 Bio-Logger Buoyancy

**Tag Materials**	**Volume [cm^3]**	**Density [g/cm^3]**	**Mass [g]**
Syntactic Foam	335	0.35	117
Epoxy	85.0	1.14	96.9
Batteries	75.8	2.35	178
3D Printed Shell	60.2	1.14	68.6
Suction Cup (4x)	53.6	1.22	65.4
Main PCB	13.0	1.77	23.0
VHF Beacon	12.0	2.34	28.1
Antenna (2x)	4.00	2.50	10.0
Recovery Board	3.90	3.08	12.0
Raspberry Pi Zero 2 W	3.70	3.24	12.0
Hydrophone (3x)	3.60	1.31	4.72
Silicone Cap	1.68	1.24	2.08
Magnetic Charging Port	1.46	2.73	3.98
**Assembled Bio-Logger**	**652.94**	**0.95**	**621.78**

The bio-logger will float after detaching from the whale since its density is lower than that of the surrounding salt water, which averages 1.03 $^{g}/_{cm^3}$.

6 Bio-Logger Pressure Cycles

The bio-logger and its pressure sensor were tested in a pressure tank (R10-10-10-H1150, High Pressure Equipment Company, Erie, PA, USA) over multiple cycles (Fig S9). The water pressure in the tank was raised to 5.5 MPa, simulating dives to 560 meters. With the pressure tank being automated, these cycles were repeated 100 times (Fig S10), allowing for a comparison between the tank pressure logger data and the bio-logger pressure sensor measurements. This setup also enabled the assessment of the sensor's consistency over time.

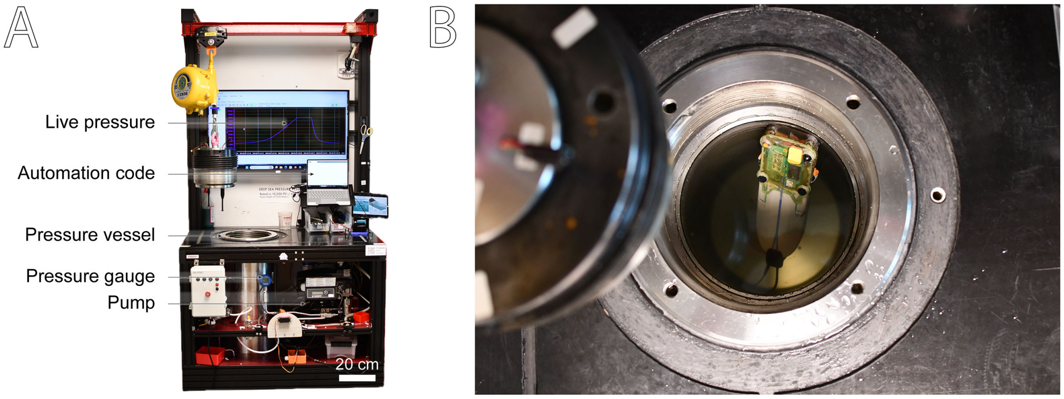

Figure S9: Pressure tank experiment. A: Overview of the pressure tank setup. B: A bio-logger after undergoing pressure cycles.

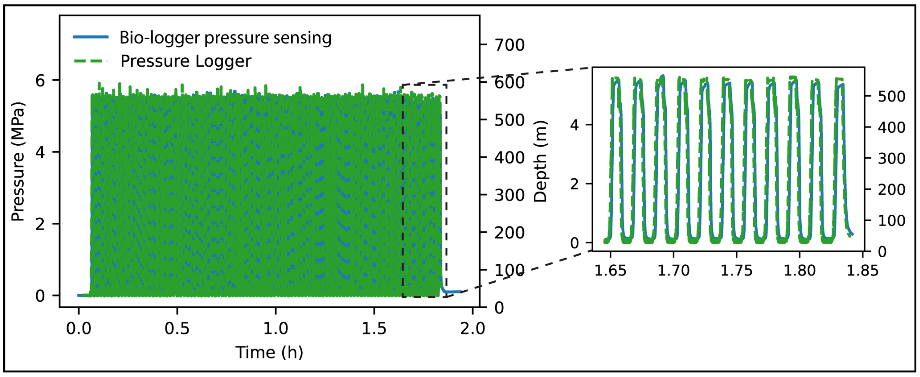

Figure S10: Sample data from the pressure tank and from the bio-logger.

7 Bio-Logger Power Budget

Table S2 provides the approximate power usage of each of the bio-logger's principal features.

Feature	Power [mW]
Single-Board Computer	1,250
Recovery Board	250
Audio ADC	210
Digital Control and Interface	150
SD Card Storage	75
Audio Analog Circuitry	50
Other Sensors	50
Power Supply Losses	150
Total	2,185

Table S2: Power consumption breakdown for the bio-logger.

8 System Resource Consumption

The system monitors and logs data about resource consumption throughout the deployment to ensure successful acquisition despite limited processing power. Fig S11 visualizes such streams including the CPU usage, RAM usage, battery voltage, and CPU temperature. These remain within reasonable levels, indicating successful resource management. The CPU usage for Core 1 demonstrates periodic spikes as audio data is written to a file every minute, while Core 3 demonstrates steady usage for audio acquisition since no other processes are allowed to use that core. The operating system is free to schedule background tasks between Core 1 and Core 2.

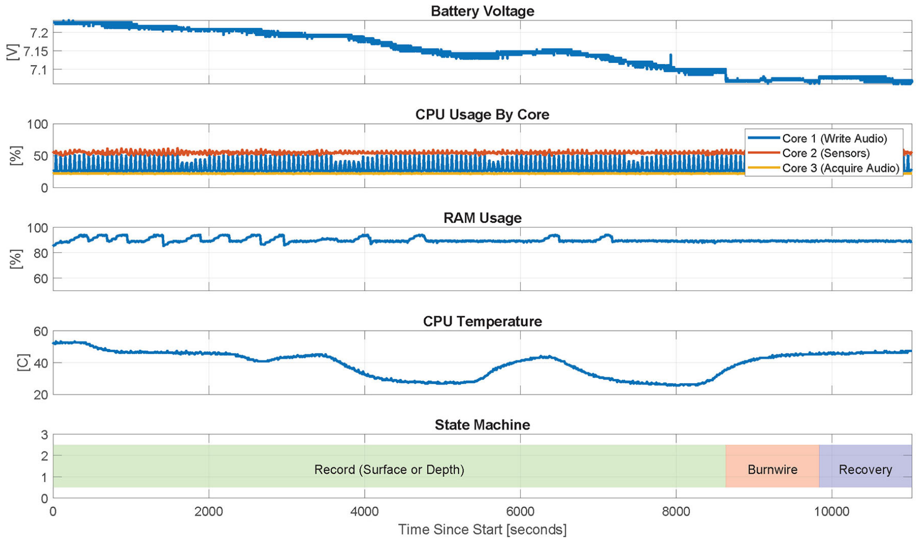

Figure S11: System resources are monitored and logged throughout a deployment to ensure proper utilization of limited resources.

9 Audio System Characterization

9.1 Introduction

In this section, the methods used to characterize hydrophone recording systems are presented. Important parameters of interest include sensitivity, frequency response, and self-noise of the system. Since the bio-logger offers an array of three sensors, it is also important to investigate channel-to-channel matching (phase and amplitude) along with crosstalk between channels, as these parameters affect the ability of the device to ideally discriminate between channels.

9.2 Underwater Characterization

A means of characterizing hydrophones and associated underwater electronics in a controlled laboratory setting is valuable as part of an overall bio-logger development and deployment program. Having a water tank facility and instrumentation in place to support general development, baseline newly manufactured devices, and evaluate units after use in the field helps to provide critical feedback to the overall project. As part of the `CETI Bio-Logger` project, a moderately sized water tank (2.4 m long × 2.4 m wide × 1.5 m deep) along with supporting filters and pumps was installed at the facility in Allston, MA, USA.

A challenge with evaluating hydrophones in any body of water is that sound waves transmitted from a projector will reflect off surfaces encountered and can complicate interpretation of the received signal. An anechoic water chamber or a very broad and deep water body is desirable, but access to these resources is often prohibitively expensive, requires boats, or is otherwise difficult in practice. However, using a pulsed technique, it is possible to measure device performance over a reasonable frequency band using a smaller volume of water [5].

In the pulsed method, a short burst of an audio tone is emitted from the projector and is received by the device under test (DUT). Depending on the tank dimensions and specific locations of the projector relative to the DUT, the tank walls, and the surface, reflections subsequently arrive at the receiving location and interfere with the measurement. However, there is a time window during which the direct wave received at the hydrophone is free from interference. For our tank with the projector and DUT located as shown in Fig S12, this interval is twice the distance between the DUT and the back wall divided by the velocity of sound in water: $(2 \times 0.7\,\mathrm{m})\left(1{,}430\,\frac{\mathrm{m}}{\mathrm{s}}\right)^{-1} \approx 980\,\mu s$.

Reflections from the side walls, bottom, and water surface arrive subsequently. The reverberations fully decay within about 100 ms. Fig S13 illustrates a typical tank response to a burst.

9.3 Sensitivity and Frequency Response

Sensitivity is defined as the ratio of the hydrophone's terminal voltage to a given applied acoustic pressure. For underwater acoustics, sensitivity is conventionally expressed in dB re 1 V/ µPa. For a digital system, we can alternatively define an overall sensitivity relative to analog-to-digital normalized counts such that a full scale *peak* digital output corresponds to 0 dB re FS/ µPa.

To measure sensitivity of a hydrophone or digital recording system in the tank, it is first necessary to set up a known sound pressure level (SPL) at the receiving location. To do this, a reference hydrophone with a known sensitivity is positioned in the tank at the reference receiving location. A 500 µs audio burst at a specific frequency is generated with either a benchtop signal generator or a PC-controlled signal generator. The signal is amplified to drive an underwater projector, and the amplitude of the burst is adjusted while monitoring the reference hydrophone's output to establish

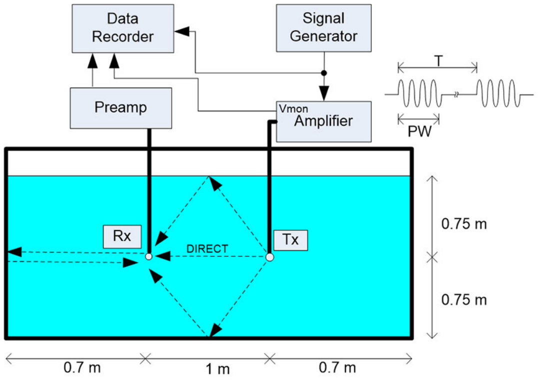

(a) Schematic of the water tank setup.

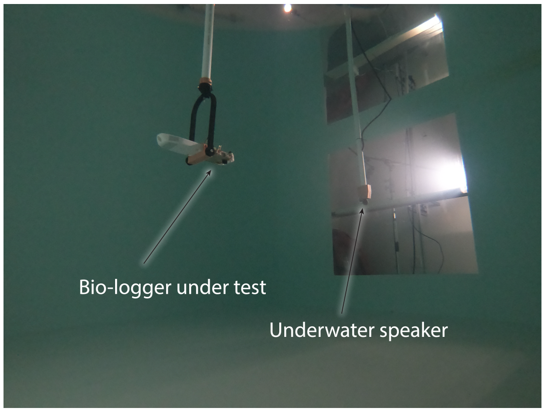

(b) Photograph of a bio-logger being held in front of a speaker within the tank.

Figure S12: Water tank setup for audio characterization.

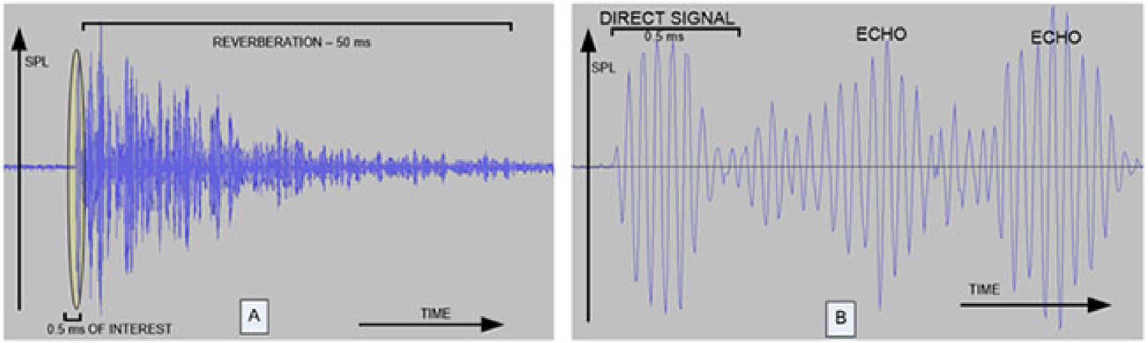

Figure S13: Burst and Reverberation. A: A 500 µs 150 dB re 1 µPa burst, and reverberations at 10 kHz as it is received by a bio-logger in the tank. The peak SPL received is approximately 1.5% of full scale. B: A zoomed-in view of the leading edge of the pulse.

a known SPL at the receiving location. This process is repeated for each frequency of interest and may be automated. The drive level for the known SPL at each frequency is recorded, and used during playback when measuring unknown devices.

For the bio-logger characterization work, we use a 140 dB re 1 µPa level and step the frequency in one third octave increments starting at 10 kHz[1] to 40 kHz. A pulse repetition rate of one second is used to ensure echoes are fully decayed. Between 40 kHz and 50 kHz we insert additional points to resolve the digital filter's rolloff with finer detail. To characterize a bio-logger, it is energized and audio recording started while it is outside the tank. It is then lowered into the tank at the receiving location and the frequency-stepped burst sequence played out. After completion of a sequence,

[1]Given the dimensions of the tank and capabilities of our projector, we have found 10 kHz to be the lowest frequency that can be measured with an uncertainty of 3 dB. It should also be noted that the pulse amplifier requires a finite rise and settling time which further constrains our low-frequency limit.

the DUT's orientation in the tank may be adjusted to explore positional effects or measure beam patterns.

Uncertainty and limitations:

In our testing, we utilized the specification sheet's nominal sensitivity of our reference hydrophone of -164 dB re 1 V/ µPa. In practice, this can vary ± 2 dB across the 10 kHz to 50 kHz band of interest. Additional uncertainty is associated with the data acquisition's system processing and estimated to be an additional ± 1 dB. This is an acceptable uncertainty for the end-use biotagging applications since factors such as placement on the focal animal dominate the overall accuracy of the levels recorded. Reduction in the uncertainty is possible, for example, by adding correction factors for reference hydrophone frequency response.

The amplitude of the test signal is limited by the capabilities of the projector and its drive amplifier. Our equipment can comfortably produce a 140 dB re 1 µPa burst at 10 kHz. Given this level and a typical DUT sensitivity of -205 dB re FS/ µPa, the recorded signal is nominally 65 dB below full scale and 30 dB above a 16-bit noise floor. This provides sufficient signal-to-noise ratio to evaluate the response, however, it only exercises a small portion of the DUT's dynamic range.

9.4 Electronic Self-Noise and Interference

The electronic self-noise of the bio-logger's audio recorder is limited principally by quantization noise, and has an RMS value of approximately 96 dB below full scale peak when measured over the full bandwidth of 40 kHz. Increasing bit-depth to 24 results in up to an 8 dB improvement in the noise floor, at which point other sources begin to dominate – these include digital noise from the bio-logger's Raspberry Pi single-board computer (SBC), input preamplifier noise, and ADC buffer noise.

When it is active, low-level ($< 0.1\%$) interference from the Wi-Fi system on the SBC is present in the audio signal and is perceptible. The SBC is located in close proximity to the audio input circuits, but on the opposite side of the main circuit board. A conductive shield over the audio circuitry is included on the board and provides some mitigation of the Wi-Fi interference. A significant portion of the interference is also associated with pickup on the wiring between the hydrophones and the board. This wiring is made short and routed away from the SBC to the extent possible. When deployed in the field, the Wi-Fi is turned off within a few minutes after the bio-logger becomes submerged, so in practice this interference is not objectionable.

The analog input and ADC circuits contribute to a small DC offset error that is typically $< 0.5\%$. There is also a low-frequency baseline drift behavior associated with the hydrophones (below 10 Hz and $< 1\%$) in the audio that is observed when the bio-logger is deployed on a whale, but not evident in static laboratory tests. The baseline level varies as a function of the focal whale's depth and motion. This artifact is reduced by the preamplifier's high-pass filter to a degree, and can be digitally filtered if needed.

9.5 Equipment List

The key instrumentation employed in our test setup is listed in Table S3. A basic configuration for manually calibrating SPL with an oscilloscope and signal generator is shown in Fig S14. When evaluating digital data captured by a bio-logger, files are uploaded after the device is removed from the tank and may be viewed and analyzed using a range of audio or mathematical software packages such as Audacity or MATLAB.

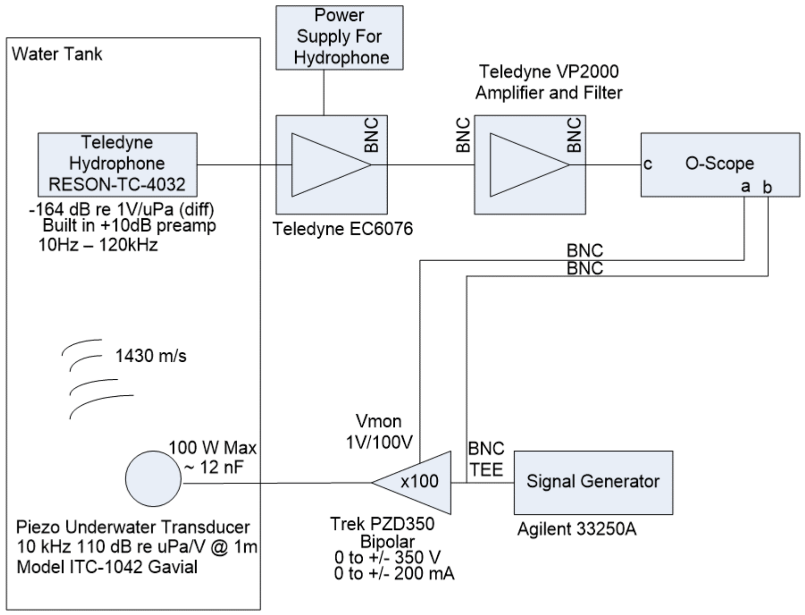

Figure S14: Instrumentation for SPL calibration.

Item	Description	Make	Model
1	Water tank (1.5 m deep x 2.4 m long x 2.4 m wide)	Custom Built	N/A
2	Omnidirectional Spherical Transducer	Gavial ITC	ITC-1042
3	HV Piezo Amplifier, 350 V, 200 mA	Trek	PZD530 (bipolar)
4	Function Generator	Agilent	33250
5	DSO, 4 channels, 100 MHz	Keysight	3014T
6	Reference Hydrophone	Teledyne Marine	TC4032
7	Hydrophone Active Input Module	Teledyne Marine	EC6076
8	Laboratory Grade Hydrophone Preamplifier	Teledyne Marine	VP2000

Table S3: Test Equipment for Hydrophone Characterization

9.6 Bio-Logger Hydrophone Array: Crosstalk, Phase, and Amplitude Matching

Good channel-to-channel isolation and phase/gain matching performance is important when utilizing the array for localization or other applications requiring differential analysis of recorded data.

Audio crosstalk due to electronic coupling between each of the bio-logger's three channels was measured by driving one input of a sample device with a 10 kHz sinusoidal signal voltage at approximately 80% of full scale using a signal generator. The remaining two channel inputs were connected to representative hydrophones having a nominal 6.8 nF capacitance. The signals were recorded and levels of the driven and non-driven channels compared using Audacity. The only

Channel	1	2	3
1	Driven	-83 dB	ND
2	-83 dB	Driven	ND
3	ND	ND	Driven

Table S4: Crosstalk between audio channels on a sample bio-logger. “ND” indicates that crosstalk is not detectable and is below the recorder’s noise floor.

	Channel		
	1	2	3
Amplitude (dBFS)	-4.03	-3.90	-4.01
Phase (degrees)	0.00 (ref)	-0.13	0.07

Table S5: Measured amplitude and phase at 10 kHz with common input on all channels, using a sample bio-logger.

measurable crosstalk occurred between channel 1 and channel 2. These channels share a dual operational amplifier, so some low-level crosstalk is expected. Results are presented in Table S4.

Phase and amplitude matching between channels, excluding the hydrophone sensors, were measured on a sample bio-logger by providing a common 10 kHz sinusoidal voltage to all channels simultaneously, capturing the data, and then evaluating the phase and amplitude differences among the three channels.

The relative amplitude values were obtained by using Audacity’s RMS measurement feature. The phase differences were obtained by comparing the sample values of the nearest zero crossing on each channel using Audacity and applying a linear interpolation relative to channel 1. Results are shown in Table S5, and reflect excellent matching between channels.

10 Hydrophone Response to Sound Pressure Level

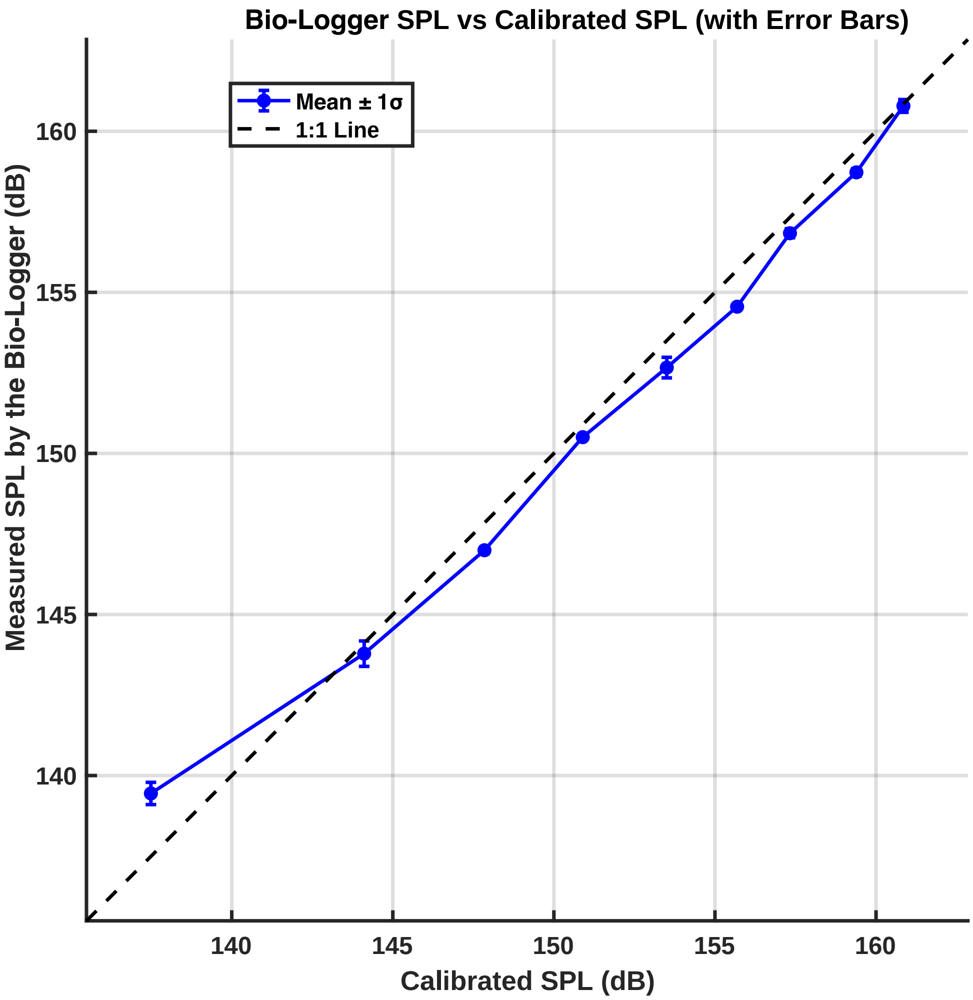

Figure S15: Estimated SPL measured at the bio-logger vs calibrated SPL.

To ensure performance of the custom hydrophones for the bio-logger, the setup in Fig S12b was repeated with a comparable bio-logger. Calibrated 10 kHz sine waves of different SPLs were played five times each; results are summarized in Fig S15. This confirmed linearity across a range of SPLs. Note that equipment limitations limited the ability to project greater SPLs, and 136 dB was near to the noise floor of the setup.

11 APRS Characterization in Dominica

On January 11th 2024, tests of the recovery board's ability to receive GPS and transmit APRS while floating in the ocean were conducted one mile off the west coast of Dominica. During the 91 minutes in the water, 91 messages were transmitted which means the bio-logger maintained GPS lock throughout the entire test. Out of the 91 APRS messages transmitted, 28 made their way to i-gates on the island ($\approx 30\%$).

On February 29th 2024, APRS range tests were performed by having the bio-logger floating in a bucket of seawater on a catamaran, at approximately 0.5 m above the ocean surface, while following whales to the north of the island as seen in Fig S16A. Over the course of 14 hours and 20 minutes, 860 messages were expected and all were successfully transmitted. There was a slightly worse probability of a transmission being received compared to the January test, with only 162 messages received ($\approx 19\%$). During this test, the farthest received message traveled ≈ 54.5 km to the antenna at the south of the island as seen in Fig S16B. This antenna, which sits high on the mountain, has an expected range of ≈ 50 km. This range test was repeated on March 3rd 2024, with the farthest message received from ≈ 73 km from the receiving radio antenna.

In addition to testing range and capabilities of the recovery board built into the bio-loggers, a recovery board has also been mounted to each of the two Project CETI boats in operation off the coast of Dominica since November 2023. APRS-IS has received over 30,000 APRS messages from these two vessels to date. Fig S16C depicts the expected probability of a message transmitted from these recovery boards being received by an i-gate within 20 km of the West coast of Dominica. There are too few data points outside our normal range of operation to quantify coverage farther than 20 km off shore. Note that these tests simulate a best-case scenario for a bio-logger in a recovery situation; during real deployments, the GPS and VHF antennae may be partially covered by water, decreasing the range and the likelihood that messages are received.

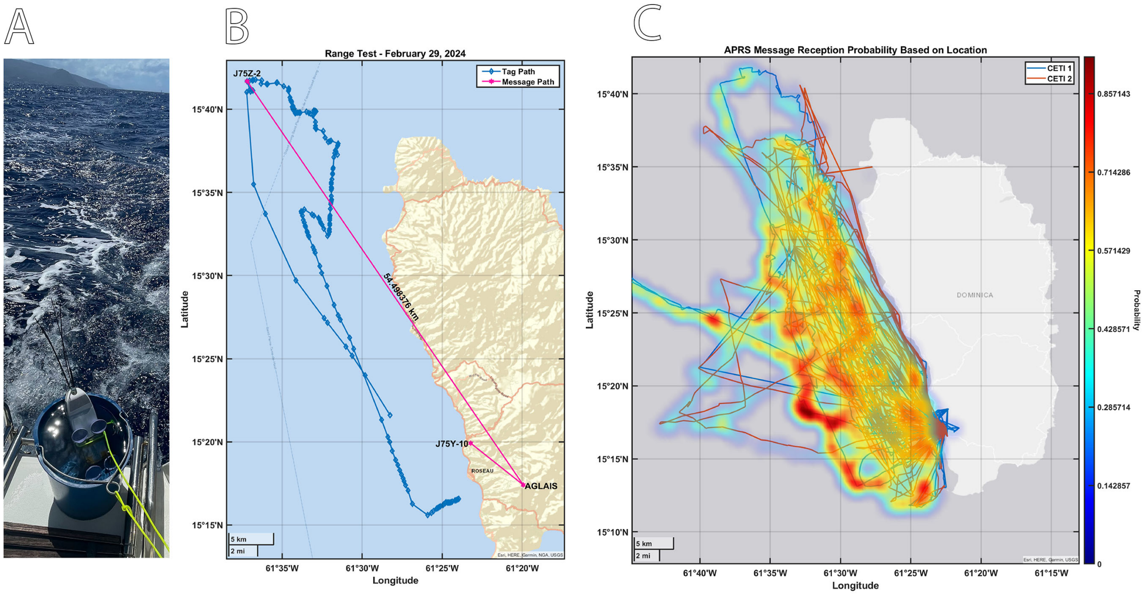

Figure S16: APRS Messages. A: The bio-logger floats in a bucket with antennae 0.5 m above the ocean surface during the tagging and tracking effort on February 29th 2024. B: APRS position data received by i-gates from the bio-logger in (A), with the farthest received message path highlighted. C: Probability of an APRS message being received by an island i-gate, based on data from boat-based recovery boards.

12 Computational Fluid Dynamics (CFD)

Simulations were performed to investigate the dynamical properties of the bio-logger. These were conducted using AirShaper [6], which is a cloud-based service. Configuration parameters for the simulation are presented in Table S6. Results are presented in Table S7 and Fig S17.

Table S6: Setup Parameters for the CFD Simulation

**Parameter**	Value
**Mesh Size**	1 million cells
**Fluid**	Water
**Fluid speed**	1.7 m/s (an average whale swimming speed)

Table S7: Results of the CFD Simulation

**Quantity**	Value
**Surface Area**	0.004 m^2
**Planform Area**	0.029 m^2
**Drag Coefficient Cd**	0.378 [-]
**Lift Coefficient Cl**	0.521 [-]

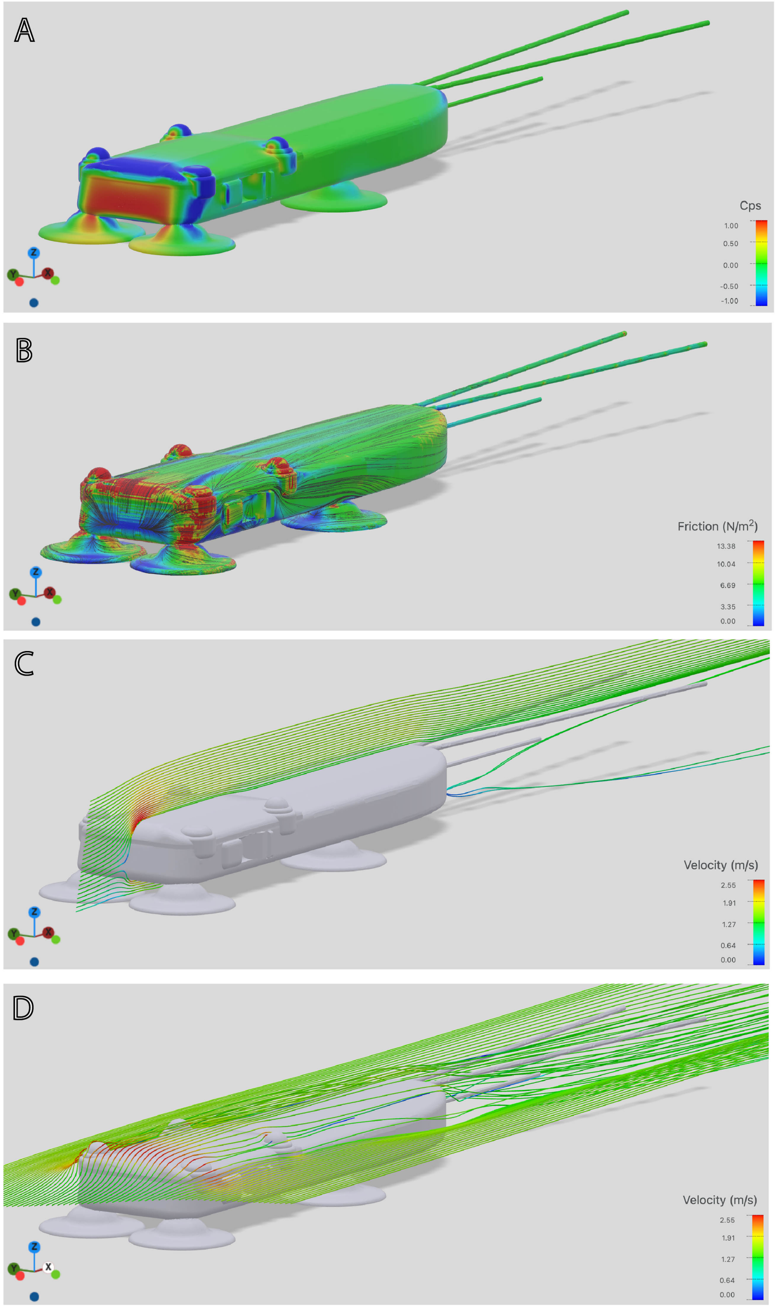

Figure S17: Results of the Computational Fluid Dynamics simulation. A: Surface Pressure. B: Surface Friction. C: Vertical Streamlines. D: Horizontal Streamlines.

13 Bill of Materials

Bills of materials are presented below for fabricating the bio-logger. General components are listed in Table S8. The recovery board PCB is then considered in Table S9, and the main PCB is considerd in Table S10.

13.1 General Components

Table S8 presents raw materials and components for the bio-logger systems. Costs such as assembly, machining, or molding are not included.

Category	Item	Part Number or Material	QTY	Price per Unit	Price per Bio-Logger
**Mechanical**	Syntactic Foam	MZ-22, ESS	1	$106.42	$106.42
	Top Shell	Polyjet VeroClear, Stratasys	1	$29.00	$29.00
	Bottom Shell	Polyjet VeroClear, Stratasys	1	$71.00	$71.00
					**Subtotal: $206.42**
**Electrical (Main)**	Batteries	6767100-1C, AA Portable Power Corp	2	$29.95	$59.90
	Single Board Computer	Raspberry Pi Zero 2 W	1	$21.95	$21.95
	Recovery Board	Custom made (see Table S9)	1	$47.86	$47.86
	Main PCB	Custom made (see Table S10)	1	$355.74	$355.74
	RF Shielding	8912K12, McMaster	1	$4.82	$4.82
					**Subtotal: $490.27**
**Satellites**	External Connector	1528-5467-ND, Digikey	2	$7.95	$15.90
	VHF Tracker	F1840B, ATS	1	$185.00	$185.00
	GPS Antenna	Custom made	1	$4.96	$4.96
	APRS Antenna	Custom made	1	$4.96	$4.96
	Burnwire	Custom made	2	$8.21	$16.42
	Suction Cups	Custom made	4	$3.50	$14.00
					**Subtotal: $241.24**
**Assembly**	Potting resin for electronics	832C, MG Chemicals	0.25	$51.56	$12.89
	UV curable glue	Krazy Glue	0.5	$10.31	$5.16
	Glue for silicone rubber	Sil-Poxy, Smooth-on	0.05	$38.10	$1.91
					**Subtotal: $18.10**
**Audio**	Accoustically transparent rubber	PR-1547, PPG Industries	0.05	$538.13	$26.91
	Piezo sphere	Piezo Hannas	3	$300.00	$900.00
					**Subtotal: $926.91**
**Total**					**Total: $1,882.94**

Table S8: A bill of materials for the bio-logger's components (excluding costs such as assembly, machining, or molding services)

13.2 Printed Circuit Boards and Other Electronic Components

Description	Manufacturer Part	Quantity	Price per Unit	Price per Board
Misc Resistors	-	21	-	$0.29
Misc Capacitors	-	23	-	$2.28
Coin Cell Battery	MS621FE-FL11E	1	$2.19	$2.19
Diode	SS14L	1	$0.06	$0.06
Blue LED	16-213/BHC-ZL1M2B6Y/3T	1	$0.30	$0.30
Red LED	16-213/SDRC/S530-A3/TR8	1	$0.32	$0.32
TVS Diode	LESD3Z5.0CMT1G	1	$0.13	$0.13
U.FL Connector	1909763-1	2	$0.23	$0.46
Ferrite Bead	BLM18PG121SN1D	1	$0.04	$0.04
Ferrite Bead	MPZ1608D300BTD25	1	$0.10	$0.10
N-Ch MOSFET	DMN65D8L-7	2	$0.17	$0.33
P-Ch MOSFET	SSM3J332R,LF	1	$0.29	$0.29
VHF Module	DRA818V	1	$14.24	$14.24
GPS Module	NEO-M9N-00B	1	$19.95	$19.95
MCU	STM32U575VGT6	1	$6.30	$6.30
32.768kHz Xtal	X321532768KGD2SI	1	$0.19	$0.19
16MHz Xtal	NX2520SA-16MHZ-STD-CSW-5	1	$0.38	$0.38
**Total**				**$47.86**

Table S9: A bill of materials for the recovery board PCB (excluding costs such as assembly services)

**Description**	**Manufacturer Part**	**QTY**	**Price per Unit**	**Price per Board**
Misc Resistors	-	140	-	$1.95
Misc Capacitors	-	134	-	$12.23
Current Sense Resistor	WSK2512R0250FEA	1	$1.12	$1.12
Feed-through Capacitors	NFM21PC104R1E3D	8	$0.16	$1.28
Misc Transistors	-	22	-	$5.21
LED0805-R-RD	17-21SYGC/S530-E1/TR8	1	$0.14	$0.14
LED0805-RD	D-R080508L3-KS2	1	$0.10	$0.10
LED0805-RD_YELLOW	17-21UYC/S530-A3/TR8	1	$0.21	$0.21
LED0805-RD_GREEN	0805G Green	1	$0.09	$0.09
3.3 v Coin Cell	MS621FE-FL11E	1	$2.19	$2.19
200 mA Resettable Fuse	MF-PSHT020KX-2	1	$1.07	$1.07
500 mA Resettable Fuse	0ZCH0050FF2G	1	$0.16	$0.16
600 Ohm Ferrite Bead	HZ1005K601TFB01	9	$0.07	$0.60
Magnetic Connector	Adafruit 5467	2	$7.95	$15.90
Depth Sensor	Keller 4LD	1	$114.30	$114.30
4.7 µH Inductor	HCM1A4020V2-4R7-R	4	$0.96	$3.84
22uH Inductor	VLS4012CX-220M-1	1	$0.38	$0.38
Audio 2Ch JFET OpAmp	AD8642ARZ-REEL7	2	$7.62	$15.24
2Ch Differential OpAmp	ADA4940-2ACPZ-R7	2	$8.15	$16.30
FPGA	XC3S200A-5VQG100C	1	$52.49	$52.49
24-bit ADC	AD7768-4BSTZ	1	$27.89	$27.89
4.096V Ref	ADR4540ARZ	1	$7.31	$7.31
Vref Buffer OpAmp	ADA4841-1YRZ	1	$5.38	$5.38
1.2 v Linear Reglator	ADP151AUJZ-1.2-R7	1	$1.53	$1.53
IMU	BOSCH HILLCREST BNO086	1	$16.85	$16.85
USB Switch	MAX4907ELA+T	3	$1.43	$4.30
I/O Expander	PCA9674ABS,118	1	$1.80	$1.80
TVS Diode	D3V3F4U6S-7	2	$0.27	$0.54
BMS	DS2778G	1	$9.57	$9.57
2A Resettable Fuse	MINISMDC200F/16-2	1	$3.09	$3.09
3.3 V Linear Reglator	ADP7118AUJZ-3.3-R7	2	$2.78	$5.56
Temperature Sensor	ADT7461ARZ-REEL	1	$3.97	$3.97
RTC	DS1374U-33+T&R	1	$4.82	$4.82
SI1865DDL-T1-GE3	SI1865DDL-T1-GE3	1	$0.04	$0.04
Buck Regulator	MP1495SGJ-P	3	$2.23	$6.69
-5V Buck Regulator	MAX17579	1	$4.36	$4.36
Ambient Light Sensor	LTR-329ALS-01	1	$1.02	$1.02
Comparator	LMV393IDR	1	$0.62	$0.62
098.304 MHz Clock	HSM613-098.304M	1	$4.34	$4.34
32.768 kHz XTAL	Q13FC1350000400	1	$1.26	$1.26
**Total**				**$355.74**

Table S10: A bill of materials for the main PCB (excluding costs such as assembly services)

14 Comparison to Existing Bio-Loggers

		CETI Bio-Logger	DTAG [7]	Acousonde 3B [8]	CATS Tag [9]
Audio	Included	✓	✓	✓	Add-on
	Channels	1-4	1-4	1-2	1
	Rate [kSPS]	192	192	232	192
	Bit Depth	24	24	*	*
	Compression	flac	X3	*	*
IMU		✓	✓	✓	✓
Ambient Light		✓	✓	✓	✓
Depth		✓	✓	✓	✓
GPS		✓	✓	-	Add-on
Open Source		✓	-	-	-
Architecture		Single-Board Computer (Linux OS)	Microcontroller *	* *	* *
Storage [GB]		256	6.6	128	*
Recovery		VHF & APRS	VHF	VHF & Strobe	VHF
Dimensions [cm]		29.4 x 8 x 5	≈ 1 liter	22.4 x 7.8 x 4.1	6.0 x 3.5 x 2.5 +
Weight [g]		700	300	362	270 +
Price		$1,882.94 **	*	*	$2,313.00 +

* Data not publicly available
** Price of components (excludes costs such as assembly, machining, or molding services)
\+ Minimum (not including any add-ons)

Table S11: Comparison of the `CETI Bio-Logger` to existing bio-loggers.

References

[1] Bandy J, Vincent N. Nutrition Label Template for Dataset Documentation; 2021. Available from: https://www.overleaf.com/latex/templates/nutrition-label-template-for-dataset-documentation/gxzpbfmncyfp/.

[2] Gebru T, Morgenstern J, Vecchione B, Vaughan JW, Wallach HM, III HD, et al. Datasheets for Datasets. CoRR. 2018;abs/1803.09010.

[3] Bell MA, Becker KP, Wood RJ. Injection Molding of Soft Robots. Advanced Materials Technologies. 2022;7(1):2100605.

[4] Hernandez AM, Sandoval JA, Yuen MC, Wood RJ. Stickiness in shear: stiffness, shape, and sealing in bioinspired suction cups affect shear performance on diverse surfaces. Bioinspiration & Biomimetics. 2024;19(3):036008.

[5] Levin PA. Calibration of Hydrophones. Bruel and Kjaer Technical Review. 1973;.

[6] AirShaper. AirShaper CFD; Accessed 2024-07-12. https://airshaper.com/.

[7] DTAG: A Digital Acoustic Recording Tag; Accessed 2024-12-18. https://www2.whoi.edu/site/marinemammalbehaviorlab/dtag/.

[8] Acousounde B3; Accessed 2024-12-18. https://www.acousonde.com/downloads/Acousonde3B_Brochure.pdf.

[9] CATS. CATS tag; Accessed 2024-03-18. https://cats.is/.
